# Comparative Genomic Analyses of Multiple *Pseudomonas* Strains Infecting *Corylus avellana* Trees Reveal the Occurrence of Two Genetic Clusters with Both Common and Distinctive Virulence and Fitness Traits

**DOI:** 10.1371/journal.pone.0131112

**Published:** 2015-07-06

**Authors:** Simone Marcelletti, Marco Scortichini

**Affiliations:** 1 Consiglio per la ricerca in agricoltura e l’analisi dell’economia agraria (C.R.A.)-Centro di Ricerca per la Frutticoltura, Via di Fioranello 52, I-00134, Roma, Italy; 2 Consiglio per la ricerca in agricoltura e l’analisi dell’economia agraria (C.R.A.)-Unità di Ricerca per la Frutticoltura, Via Torrino 3, I-81100, Caserta, Italy; Niels Bohr Institute, DENMARK

## Abstract

The European hazelnut (*Corylus avellana*) is threatened in Europe by several pseudomonads which cause symptoms ranging from twig dieback to tree death. A comparison of the draft genomes of nine *Pseudomonas* strains isolated from symptomatic *C*. *avellana* trees was performed to identify common and distinctive genomic traits. The thorough assessment of genetic relationships among the strains revealed two clearly distinct clusters: *P*. *avellanae* and *P*. *syringae*. The latter including the pathovars *avellanae*, *coryli* and *syringae*. Between these two clusters, no recombination event was found. A genomic island of approximately 20 kb, containing the *hrp/hrc* type III secretion system gene cluster, was found to be present without any genomic difference in all nine pseudomonads. The type III secretion system effector repertoires were remarkably different in the two groups, with *P*. *avellanae* showing a higher number of effectors. Homologue genes of the antimetabolite mangotoxin and ice nucleation activity clusters were found solely in all *P*. *syringae* pathovar strains, whereas the siderophore yersiniabactin was only present in *P*. *avellanae*. All nine strains have genes coding for pectic enzymes and sucrose metabolism. By contrast, they do not have genes coding for indolacetic acid and anti-insect toxin. Collectively, this study reveals that genomically different *Pseudomonas* can converge on the same host plant by suppressing the host defence mechanisms with the use of different virulence weapons. The integration into their genomes of a horizontally acquired genomic island could play a fundamental role in their evolution, perhaps giving them the ability to exploit new ecological niches.

## Introduction

The European hazelnut (*Corylus avellana*) is a valuable crop and is cultivated in many temperate areas of the world. In some countries, such as Turkey and Italy, its extensive cultivation began in ancient times (i.e., more than 2.000 years ago), whereas in other areas (U.S.A., Spain, France, Greece, Iran), the cultivation of this species only began during last century. During the 1980s-1990s, an emerging pseudomonad caused severe economic losses both in northern Greece and central Italy [1, 2]. The causal agent of the bacterial canker of the European hazelnut was initially identified as *Pseudomonas syringae* pv. *avellanae* [3, 4]. Subsequently, the pathogen was elevated to the species-genomospecies level and named *P*. *avellanae* [5, 6]. Conversely, based on the MLSA approach, it was again placed in the *P*. *syringae* complex [7]. However, further taxonomic assessments strongly supported the existence of a well-demarcated *P*. *avellanae* genomospecies (i.e., genomospecies 8), including strains isolated in Greece and Italy, as well as of strains isolated only in Italy, belonging to *P*. *syringae* and classified as *P*. *s*. pv. *avellanae* [8, 9]. This distinctiveness was also further confirmed by Berge *et al*. [10] which included *P*. *avellanae* in the phylogroup 1 (i.e., PG 01b) and *P*. *s*. pv. *avellanae* in the phylogroup 2 (i.e., PG 02b). These two distinct groups of strains represent a case of pathogenic convergence onto the same host plant [11, 12]. They can also be distinguished by repetitive sequence-PCR, 16S-23S rRNA genotyping, and MLST analysis [13–16].

Additional studies performed in the major areas of European hazelnut cultivation in Italy revealed the presence of another pathogenic pseudomonad causing twig dieback and branch cankers: *P*. *s*. pv. *coryli* [17, 18]. This pathogen was also isolated in Germany [19]. Finally, other surveys performed in the same areas of Italy where the other three pseudomonads were isolated, identified the polyphagous *P*. *s*. pv. *syringae* which has the capacity to cause twig dieback [20, 21]. Genomically, *P*. *s*. pv. *avellanae*, *P*. *s*. pv. *coryli* and the *P*. *s*. pv. *syringae* strains isolated from the European hazelnut are very closely related and belong to the same genomospecies, namely genomospecies 1 [9, 22].

Epidemiological studies and field surveys have demonstrated differing levels of symptoms severity caused by the four pseudomonads to *C*. *avellana*. *P*. *avellanae* and *P*. *s*. pv. *avellanae* are the most dangerous because they can cause extensive twig wilting and dieback, canker formation along the trunk and plant death. *P*. *s*. pv. *coryli* and *P*. *s*. pv. *syringae* appear to be less aggressive and mainly cause twig dieback ((S1 Table and S1 Fig)). *P*. *avellanae* has also been shown to migrate systemically within the plant, and it can infect wild *C*. *avellana* trees grown near infected hazelnut orchard [14, 23]. In addition, the occurrence of endophytic and potentially pathogenic *P*. *syringae* strains was observed in symptomless wild trees of *C*. *avellana* trees [24].

The fact that four distinct pseudomonads belonging to two distinct genomospecies, namely *P*. *avellanae* and *P*. *syringae*, can infect the same host plant, prompted us to genomically investigate some of their characteristics to possibly identify the common and peculiar traits that enable them to colonize and infect *C*. *avellana*. Comparative genomics were already successfully applied for plant pathogenic pseudomonads to elucidate the basic features involved in the pathogenicity, virulence and environmental fitness of the strains analysed [25–29]. To accomplish this aim, we reciprocally compared the draft genomes of the nine strains of these two genomospecies, focusing the analyses on genes/proteins involved in the infection process and in the *in planta* fitness. In addition, particular attention was devoted to clearly define the distinctiveness of the two clusters using multiple taxonomic approaches. In fact, bacterial speciation is intimately linked to their ecological specialization [30] and the pathogenic convergence of some strains of the species to a particular cultivated host plant can represent just one facet of their more common behaviour and presence in other niches. Strains of these two clusters have been considered to belong to the *P*. *syringae* complex [12, 31, 32]. Our analyses, performed with several different, robust clustering approaches, demonstrated that *P*. *avellanae* is distinct from the *P*. *syringae* pathovars *avellanae*, *coryli* and *syringae* strains. This distinction is further supported by the absence of recombination between the two clusters, which have remarkably different repertoires of type III secretion system effectors, possibly due to the different evolutionary trajectories of the two genomospecies. A genomic island, containing the *hrp/hrc* type III secretion system, was found in all strains, whereas some features related to virulence as well as to the *in planta* fitness such as the presence of mangotoxin, yersiniabactin and production of ice nuclei genes were found at different levels in the two genomospecies.

## Materials and Methods

### Library preparation, genome sequencing, assembly, annotation and comparison

For genomic sequencing, the DNA of *P*. *avellanae* CRAFRU ec1, CRAFRU ed2, CRAFRU ee3 and *P*. *s*. pv. *syringae* CRAFRU 11 and CRAFRU 12, was prepared as described elsewhere [8, 9, 27]. DNA was sequenced using Illumina Genome Analyzer IIx (Illumina, San Diego, CA, USA), at the Istituto di Genomica Applicata (Udine, Italy). In order to remove contaminants and adaptors, quality check of reads was performed using the Extended Randomized Numerical alignEr (ERNE) filters, a string alignment package to provide set of tools to handle short reads (http://www.erne.sourceforge.net). Paired reads of 100 nts were assembled into contigs using the *de novo* (i.e. without using a reference genome) assembly option of the CLC genomic workbench (CLC-bio, Aarhus, Denmark) by setting the default parameters. Contigs sequences were scanned for ORFs by GLIMMER, version 3.02. [33] which had been previously trained on the complete genome sequences of *P*. *s*. pv. *tomato* DC 3000 (NC_004578.1), *P*. *s*. pv. *phaseolicola* 1448A (NC_005773.), *P*. *s*. pv. *syringae* B728a (NC_007005). Genome comparisons were also performed with previously sequenced genomes of *P*. *avellanae* BPIC 631 (ATDK00000000), *P*. *s*. pv. *avellanae* CRAPAV 013 (AKCJ00000000), *P*. *s*. pv. *avellanae* CRAPAV 037 (AKCK00000000), *P*. *s*. pv. *coryli* NCPPB 4273 (AWQP00000000) and *P*. *cannabina* pv. *alisalensis* BS91 (ID 2516653056). The putative proteins were annotated against the RefSeq database using *ad hoc* PERL scripts for recursive BlastX searches [34] and MUMmer, version 3.20 software [35]. The type III secretion system effectors were identified by means of an *ad hoc* script through tBlastn and the website database http://www.pseudomonas-syringae.org., with the following cutoff: evalue e-10, length hit > 60%. A dendrogram of effector relationships, based on their presence/absence in the genomes, was built using the UPGMA algorithm and the web tool Tree drawing, available at http://www.pubmlst.org website. *P*. *cannabina* pv. *alisalensis* BS91 was included in the analyses as outgroup. Bacteriocins were searched through the web tool Bagel available at htpp//:wwwbagel2.molgenrug.nl.

### Average Nucleotide Identity (ANI) and tetranucleotide frequency correlation coefficients (TETRA) analyses

To evaluate the taxonomic relationships of the nine *Pseudomonas* strains infecting *C*. *avellana*, the average nucleotide identity (ANI) and tetranucleotide frequency correlation coefficients (TETRA) analyses were performed. The analyses of sequences for the determination of their relatedness according to ANI and TETRA were performed with the software JSpecies [36]. ANI was calculated using the MUMmer algorithm implementation, version 3.20 (i.e., ANIm) [35]. TETRA was used as an alignment-free genomic similarity index as oligonucleotide frequencies carry a species-specific signal. The use of a tetranucleotide usage pattern has been shown to be a good compromise between signal strength and need computational power [36]. Pairwise comparison between genomes is performed by plotting the corresponding tetranucleotide frequency and then obtaining a regression line. ANI analysis has recently been proposed as a new standard for inferring robust taxonomic relationships between bacterial species based on genome comparison and it has been assumed that values of 95% or 95–96% for ANI correspond to the 70% of the DNA-DNA hybridization reassociation value for demarcating bacterial species.

### Genetic relationships and evolutionary history based on MLSA and split networks

To further evaluate the genetic relationships of the nine strains, a multilocus sequence typing analysis (MLSA) and a neighbor-net network were built using four housekeeping genes (*gapA*, *gltA*, *gyrB*, and *rpoB*,), for a total of 6.846 nucleotides. For MLSA, the maximum likelihood (ML) analysis was inferred with PhyML version 3.0 [37], with 100 bootstrap replicates. To select the best fit model for ML analysis, we used a PhyML test procedure implemented in the R package APE [38]. JC69 was used as best substitution model. The corresponding tree was visualized using FigTree software, version 1.1.2 (http://www.tree.bio.ed.ac.uk/software/figtree/). The genetic relationships among the strains was also assessed using consensus networks [39]. A data set containing ortholog alignment was prepared using a multistep procedure based on several *ad hoc* PERL scripts. First, the predicted protein sequences of all genomes were analyzed for the identification superfamilies of homologs by a procedure based on reciprocal smallest distance algorithm [40]. Subsequent application of the branch clustering algorithm BranchClust [41], allowed delineation of families of orthologs within superfamilies containing one or more paralogous gene families. Families for analysis were selected by excluding those that did not consist of one protein per genome or that contained more than one protein per genome. Those that did not pass a quality check (i.e., with a mean < 0.7 or a standard deviation < 0.05 in the identity values calculated between all pairs of proteins) and those that contained at least one sequence consisting of more than 4% of the positions as internal indels were also excluded. In total, 2.812 gene sequence alignments, spanning 1.977.984 nucleotide sites, were selected for the phylogenetic analysis. The trees from each individual DNA sequence alignment were obtained by recursively running PhyML using LC as a substitution model and Nearest Neighbor Interchange (NNI) for the tree topology estimate. From the 2.812 gene sequence alignment ML trees, a consensus network, regarding the nine pseudomonads, was obtained with Splits Tree 4, using a mean network construction [39]. These networks display edges that occur in a proportion of the gene trees above a threshold value. The presence of reticulation in the network indicates contradictory evidence in the grouping. The core gene sequences were also concatenated (a total of 1.977.984 nucleotides) to obtain a single large alignment. The alignment was then submitted to Neighbor-Network analysis with Splits Tree 4 using the neighbor-joining (NJ) algorithm with the Hamming distance method for building the consensus network and neighbor-net trees. Bootstrap analysis was performed with 100 replications by using the same software. *P*. *cannabina* pv. *alisalensis* BS91 was included in the analyses as an outgroup.

### Recombination, coalescence, gene flow and adaptive divergence

A first assessment of the recombination events between the nine pseudomonads was inferred by checking the possible presence of reticulation both in the consensus and the neighbor-net networks described above. For the four housekeeping genes, the recombination networks were built on using Splits Tree 4 software [39]. In such evolutionary networks, reticulation indicates possible events of recombination among strains [39]. Afterword, to additionally evaluate possible breakpoints due to recombination in the housekeeping genes, a likelihood-based model selection procedure was applied using the Genetic Algorhitm Recombination Detection (GARD) methods package [42] available at the http://www.datamonkey.org website. The possible clonal relationships between the strains were assessed using the ClonalFrame software [43]. This method uses mulitlocus sequence data to infer clonal relationships and to verify if the strains share a common ancestor. The gene flow between the two species was analysed using the DnaSP package, version 5.10.1 [44] by assessing the four housekeeping genes. The McDonald-Kreitman (MK) test was performed with the DnaSP package version 5.10.1 software to infer adaptive divergence between the strains. The MK test evaluates whether an excess of replacement mutations versus synonymous mutations had been fixed between the two species compared with replacement and synonymous polymorphisms within each species. According to the congruence principle suggested by Tibayrenc and Ayala [45], the four housekeeping genes assessed in the MLSA were analysed (i.e., *gapA*, *gltA*, *gyrB* and *rpoD*) were analysed in each test.

### Estimated divergence time

A divergence time estimation for the most recent common ancestor shared by the two genetic clusters was performed. Analyses were carried out using an uncorrelated lognormal relaxed molecular clock in BEAST, using the version 1.6.2 package [46] with unlinked trees and substitution models to allow for recombination between loci. The HKY substitution model was used with gamma-distributed rate variation, with separate partitions for codon positions 1 + 2 and for third positions. Substitution rates were set according to previously published rates based on the split of *Escherichia coli* and *Salmonella typhimurium* [47] and the emergence of methicillin resistant *Staphylococcus aureus* [48]. Two independent Markov chains were run for 50 million generations, and the results were combined for the parameter estimates. A divergence dendrogram based on the concatenated dataset of the four housekeeping genes applied to the *E*. *coli-Salmonella* model, was built with the DensiTree software (htpp://www.cs.auckland.ac.nz).

### Genomic islands and CRISPRs assessment

To identify putative genomic islands based on conserved flanking blocks (i.e., tRNAcc), we used the interactive online software MobilomeFINDER [49] with the IslandScreen tool, available at website: http://www.mml.sjtu.edu.cn/MobilomeFINDER, as the input for the Mauve, version 2.3.1, files. The position and number of tRNAs was assessed using the ARAGORN software, available at: http://www.mbio-serv2.mbioekol.lu.se/ARAGORN with the *P*. *avellanae* BPIC 631 genome as the reference genome. The predicted genomic islands were subjected to manual validation and delineation of the probable genomic island size. The following criteria were used: presence of mobile elements such as transposases and integrases; the over-representation of virulence-related genes, genes annotated as hypothetical proteins, and/or outbreak clade-specific genes, and the presence of adjacent tRNA genes. Genomic islands longer than 5.000 nucleotides were analysed for the gene content. The RAST server was employed to compare the gene content of the genomic islands, and the SEED viewer comparative tool was utilized to graphically represent the islands [50]. The graphical representation of the genomic islands was also obtained using R statistic-based software (http://www.R-project.org). For each genome, the possible presence of clustered regularly interspaced short palindromic repeats (CRISPRs), a microbial defence system mechanism against invading phages and plasmids, was assessed using the web tool CRISPRFinder [51], available at: http://www.crispr.u-psud.fr.

## Results

### Genome sequence data

We generated new sequence data from *P*. *avellanae* CRAFRU ec1. CRAFRU ed2, CRAFRU ee3, and for *P*. *s*. pv. *syringae* CRAFRU 11 and CRAFRU 12 strains. The main genomic features of the draft genomes are shown in Table 1. The sequence of the assembly were deposited in NCBI GenBank under the following accession numbers: *P*. *avellanae* CRAFRU ec1: ATLL00000000; *P*. *avellanae* CRAFRU ed2 AYRI00000000; *P*. *avellanae* ee3: AYRJ00000000; *P*. *s*. pv. *syringae* CRAFRU 11: ATSU00000000; *P*. *s*. pv. *syringae* CRAFRU 12: ATSV00000000.

**Table 1:** General features of draft genomes for the phytopathogenic *Pseudomonas* strains causing infection to *Corylus avellana* trees assessed in this study.

Strain	Species/Pathovar	Country, year of isolation	Genome length	Proteine-coding sequences (CDSs)	Reads	No. contigs	N50	Coverage	% GC
PaveBPIC631^T^	*Pseudomonas avellanae*	Greece, 1976	5,963,015	5187	5,823,418	612	29.502	42	58.5
PaveCRAFRUec1	*Pseudomonas avellanae*	Italy, 2003	5.736.089	5351	13.639.825	547	16.957	118	59,0
PaveCRAFRUed2	*Pseudomonas avellanae*	Italy, 2007	5.651.761	5291	16.095.188	509	17.329	142	59,0
PaveCRAFRUee3	*Pseudomonas avellanae*	Italy, 1993	5.654.685	5287	16.100.501	515	17.360	142	59,0
PssCRAFRU11	*Pseudomonas syringae* pv. *syringae*	Italy, 2005	5,859,499	4901	3,381,518	180	81.543	25	59,1
PssCRAFRU12	*Pseudomonas syringae* pv. *syringae*	Italy, 2005	5,933,506	5019	3,350,210	248	50.660	27	59,4
PscNCPPB4273	*Pseudomonas syringae* pv. *coryli*	Italy, 2001	6.096.328	5489	6.398.721	75	132.772	155	59,2
PsaveCRAPAV013	*Pseudomonas syringae* pv. *avellanae*	Italy, 1992	6.165.792	5136	n.f.	389	30.917	n.f.	59,1
PsaveCRAPAV037	*Pseudomonas syringae* pv. *avellanae*	Italy, 1993	6.050.967	5078	n.f.	220	61.365	n.f.	59,2

Psave CRAPAV 013 and Psave CRAPAV 037 are the same strains previously named as ISPaVe 013 and ISPaVe 037, respectively.

^T^: pathotype strain.

### *P*. *avellanae* is a distinct genomospecies from *P*. *syringae*

**Fig 1:**
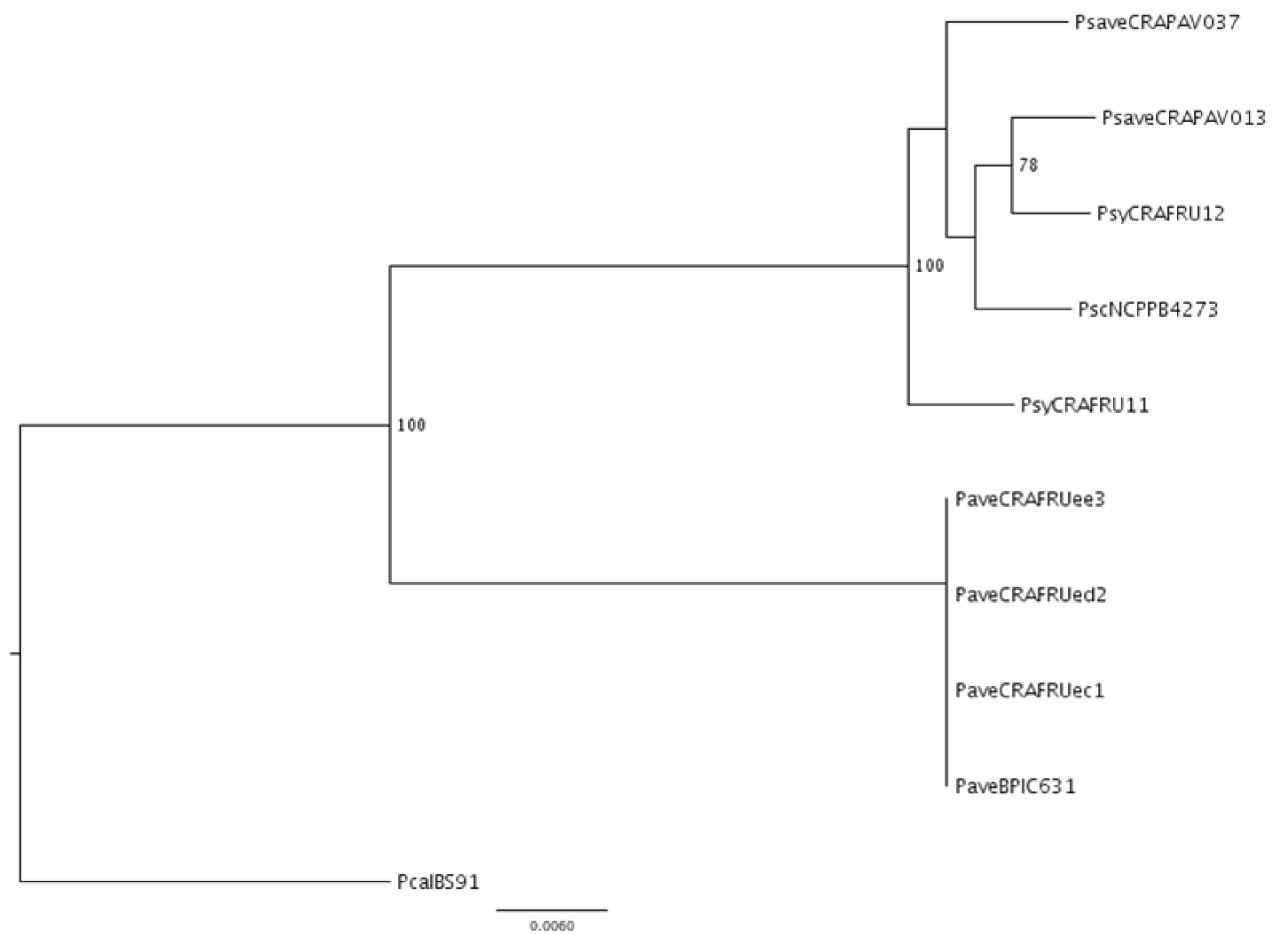
Maximum-likelihood tree based on the concatenated sequences of four housekeeping genes. Dendrogram based on MLSA analysis of *gapA*, *gltA*, *gyrB* and *rpoD*, for a total of 6.812 nucleotides regarding *Pseudomonas avellanae* and *P*. *syringae* pathovars *avellanae*, *coryli* and *syringae* strains infecting *Corylus avellana* trees. The horizontal lines show the genetic distance. The numbers at the node are support value estimated with 100 bootstrap replicates. Only bootstrap values > 75 are indicated. The scale bar indicates the number of substitutions per nucleotide position. *P*. *cannabina* pv. *alisalensis* Pcal BS91 was included in the analysis as outgroup. Strain legend is shown in Table 1.

**Fig 2:**
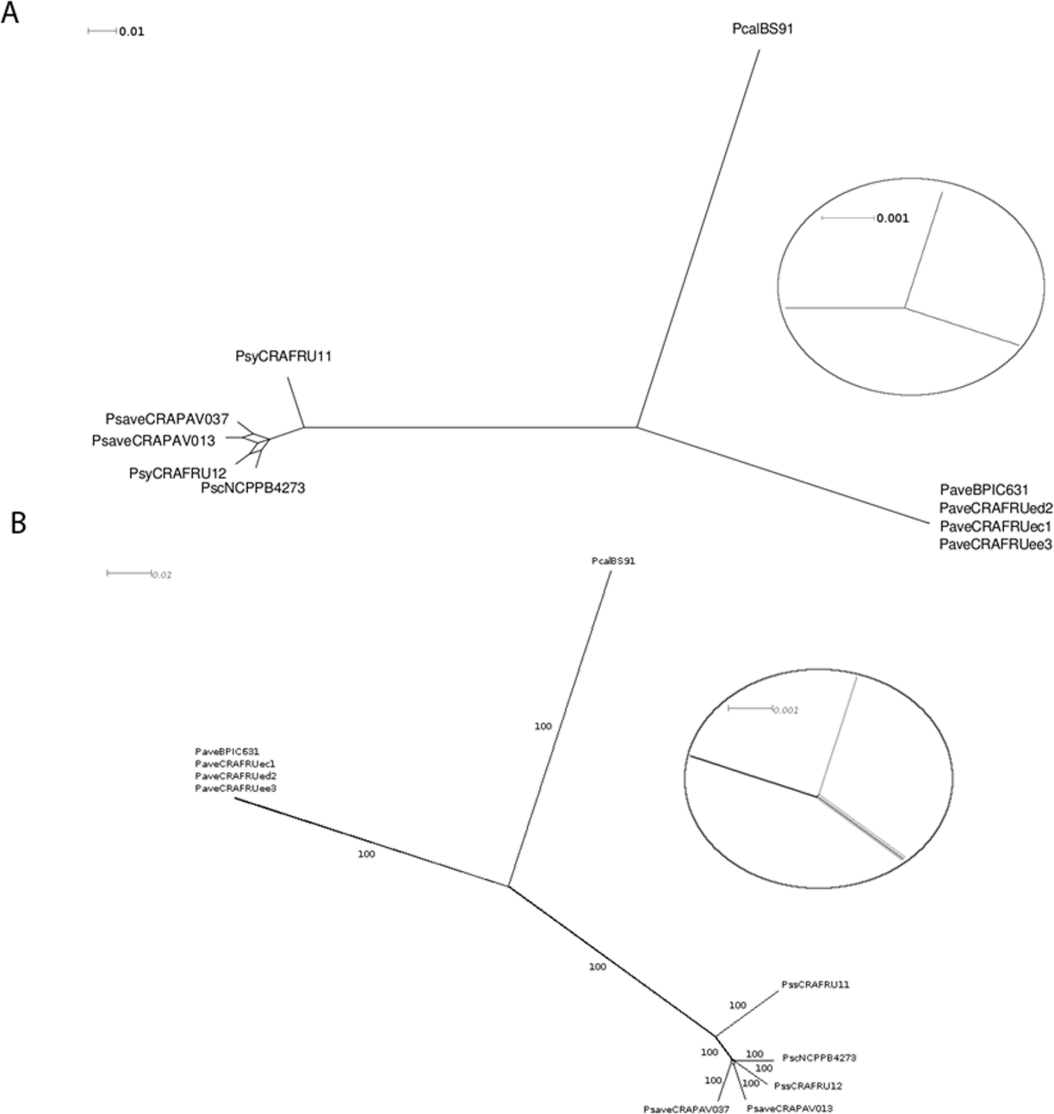
Split consensus network obtained from 2.812 trees for the nine pseudomonad strains infecting *Corylus avellana*. **A.** The tree has a cut-off value of 0.19. The scale bar indicates the number of substitutions per nucleotide position. Strain legend is shown in Table 1. No reticulation was observed between the *Pseudomonas avellanae* and *P*. *syringae* pathovar strains. **B**. Neighbor-net tree obtained from 2.812 core gene sequence alignments. The dendrogram (i.e., a total of 1.977.984 nucleotides) regards the nine pseudomonad strains infecting *Corylus avellana* trees. The scale bar indicates the number of substitutions per nucleotide position. Bootstrap values are shown at the main nodes. No reticulation was observed between the *Pseudomonas avellanae* and *P*. *syringae* pathovar strains. In both trees, *P*. *cannabina* pv. *alisalensis* Pcal BS91 was included in the analysis as outgroup. Strain legend is shown in Table 1.

The five newly sequenced genomes, together with those of the four other draft genomes of the pseudomonads infecting *C*. *avellana* trees, were cross-compared to determine their sequence similarity. The ANI value calculations, based on the MUMmer alignment of each sequence pair and the TETRA analysis are shown in Tables 2 and 3. The *P*. *avellanae* strains showed reciprocal ANI values ranging from 99.84% to 99.98%, whereas the *P*. *syringae* pathovar strains exhibited reciprocal values from 96.32% to 98.21%. When the draft genomes of the two groups were compared the reciprocal ANI values never exceeded 88% which was remarkably lower than the 95–96% used to determine a species boundary. The ML dendrogram referring to MLSA (Fig 1) and the neighbor-net network (S2 Fig), both performed with four concatenated housekeeping genes and a total of 6.846 nucleotides, indicated that the four *P*. *avellanae* strains clustered separately from the *P*. *syringae* pathovar strains as well as from the *P*. *cannabina* pv. *alisalensis* BS91 strain used as an outgroup. The *P*. *avellanae* strains clustered in a single clade, whereas each *P*. *syringae* pathovar strain exhibited a distinctive nucleotide profile. The consensus network with a cut-off value of 0,19 and based on 2.812 trees (Fig 2A) and the neighbor-net network based on 1.977.984 nucleotides (Fig 2B) also revealed that the two groups of strains clearly clustered separately with *P*. *avellanae* falling into one clade and the *P*. *syringae* pathovars exhibiting a separate bifurcation with no sign of reticulation. Collectively, these data strongly support the robust demarcation of the two groups of pseudomonads into two distinct clusters.

**Table 2:** Average nucleotide identity (ANIm) values calculated between genomes of nine *Pseudomonas* strains causing disease to *Corylus avellana* trees (see also Table 3).

ANIm									
	PssCRAFRU11	PssCRAFRU12	PsaveCRAPAV013	PsaveCRAPAV037	PscNCPPB4273	PaveBPIC631	PaveCRAFRUec1	PaveCRAFRUed2	PaveCRAFRUee3
PssCRAFRU11	—-								
PssCRAFRU12	96,32	—-							
PsaveCRAPAV013	96,58	98,21	—-						
PsaveCRAPAV037	96,59	98,06	98,16	—-					
PscNCPPB4273	96,54	98,21	98,20	98,02	—-				
PaveBPIC631	87,69	87,86	87,82	87,76	87,87	—-			
PaveCRAFRUec1	87,70	87,86	87,85	87,79	87,85	99,86	—-		
PaveCRAFRUed2	87,69	87,86	87,83	87,79	87,88	99,84	99,94	—-	
PaveCRAFRUee3	87,69	87,86	87,83	87,79	87,87	99,84	99,95	99,98	—-

**Table 3:** Tetranucleotide frequency correlation coefficients (TETRA) values calculated between genomes of nine *Pseudomonas* strains causing disease to *Corylus avellana* trees (see also Table 2).

TETRA									
	PssCRAFRU11	PssCRAFRU12	PsaveCRAPAV013	PsaveCRAPAV037	PscNCPPB4273	PaveBPIC631	PaveCRAFRUec1	PaveCRAFRUed2	PaveCRAFRUee3
PssCRAFRU11	—-								
PssCRAFRU12	0,99934	—-							
PsaveCRAPAV013	0,99917	0,99944	—-						
PsaveCRAPAV037	0,99932	0,99958	0,99953	—-					
PscNCPPB4273	0,99928	0,99968	0,99951	0,99967	—-				
PaveBPIC631	0,99213	0,99238	0,99216	0,99229	0,99215	—-			
PaveCRAFRUec1	0,99249	0,99269	0,99233	0,99250	0,99252	0,99870	—-		
PaveCRAFRUed2	0,99237	0,99262	0,99208	0,99232	0,99240	0,99848	0,99993	—-	
PaveCRAFRUee3	0,99246	0,99270	0,99222	0,99239	0,99246	0,99854	0,99993	0,99999	—-

### Recombination, coalescence, gene flow and adaptive divergence

**Fig 3:**
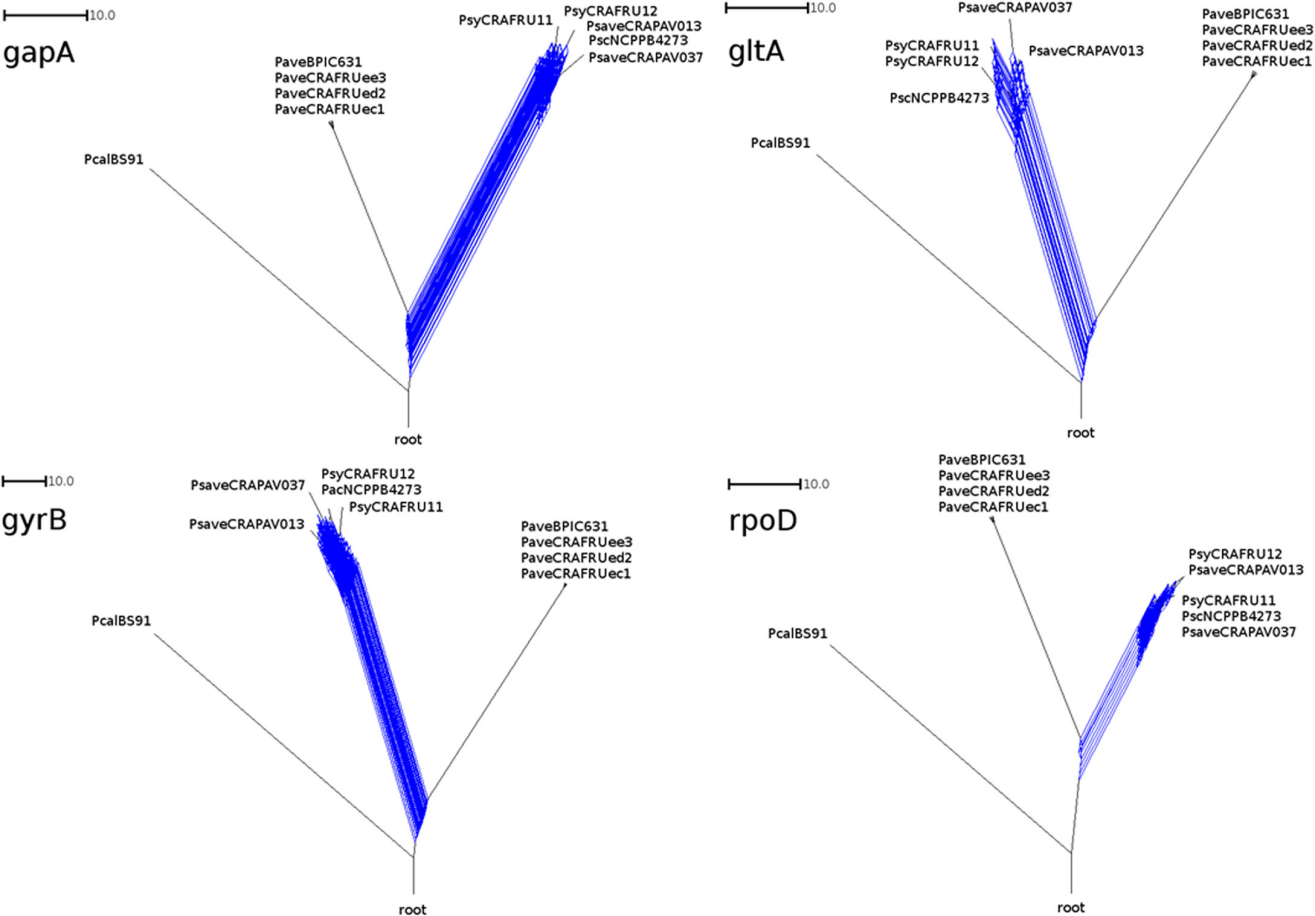
Recombination networks regarding four housekeeping genes of the nine pseudomonad strains infecting *Corylus avellana* trees. The networks were built using *gapA*, *gltA*, *gyrB*, *rpoD* genes. Strain legend is shown in Table 1. The scale bar indicates the number of substitutions per nucleotide position. Strong signal of recombination are present solely between the *Pseudomonas syringae* pathovars. *P*. *cannabina* pv. *alisalensis* Pcal BS91 was included in the analysis as outgroup.

Neither the consensus nor the neighbor-net networks built with 2.812 trees and 1.977.984 nucleotides, respectively, revealed any reticulation between the strains belonging to the *P*. *avellanae* and *P*. *syringae* clusters (Fig 2A and 2B and S2 Fig). Additionally, the recombination networks performed with the four housekeeping genes using the Split Tree 4 software did not identify any reticulation between the two groups. By contrast, signs of recombination events were evident in each of the housekeeping genes assessed here from the *P*. *syringae* pathovars *avellanae*, *coryli* and *syringae* strains infecting *C*. *avellana* (Fig 3). GARD analysis confirmed the absence of recombination between the *P*. *avellanae* and *P*. *syringae* strains. The coalescent tree obtained from the Clonal Frame (S3 Fig) revealed the same clustering as that observed with MLSA. In fact, the *P*. *avellanae* strains clustered separately from the *P*. *syringae* pathovar strains, thus revealing two distinct clonal complexes. According to the Clonal Frame analysis, the four *P*. *avellanae* strains coalesced at the same time (coalescent unit of 2.88), whereas the *P syringae* pathovars strains showed different coalescent times, with a species coalescent unit of 2.60. No recent common ancestor was found for the two clusters. The gene flow measured using the fixation index (F_ST_) and calculated with the four housekeeping genes ranged from 0.882 to 0.907. These high values confirmed the distinctiveness of the two groups. Additionally, the McDonald-Kreitman (MK) tests for adaptive divergence between *P*. *avellanae* and the *P*. *syringae* pathovars *avellanae*, *coryli* and *syringae* strains run on the four housekeeping genes did not reveal any evidence of positive selection.

### Estimated divergence time

**Fig 4:**
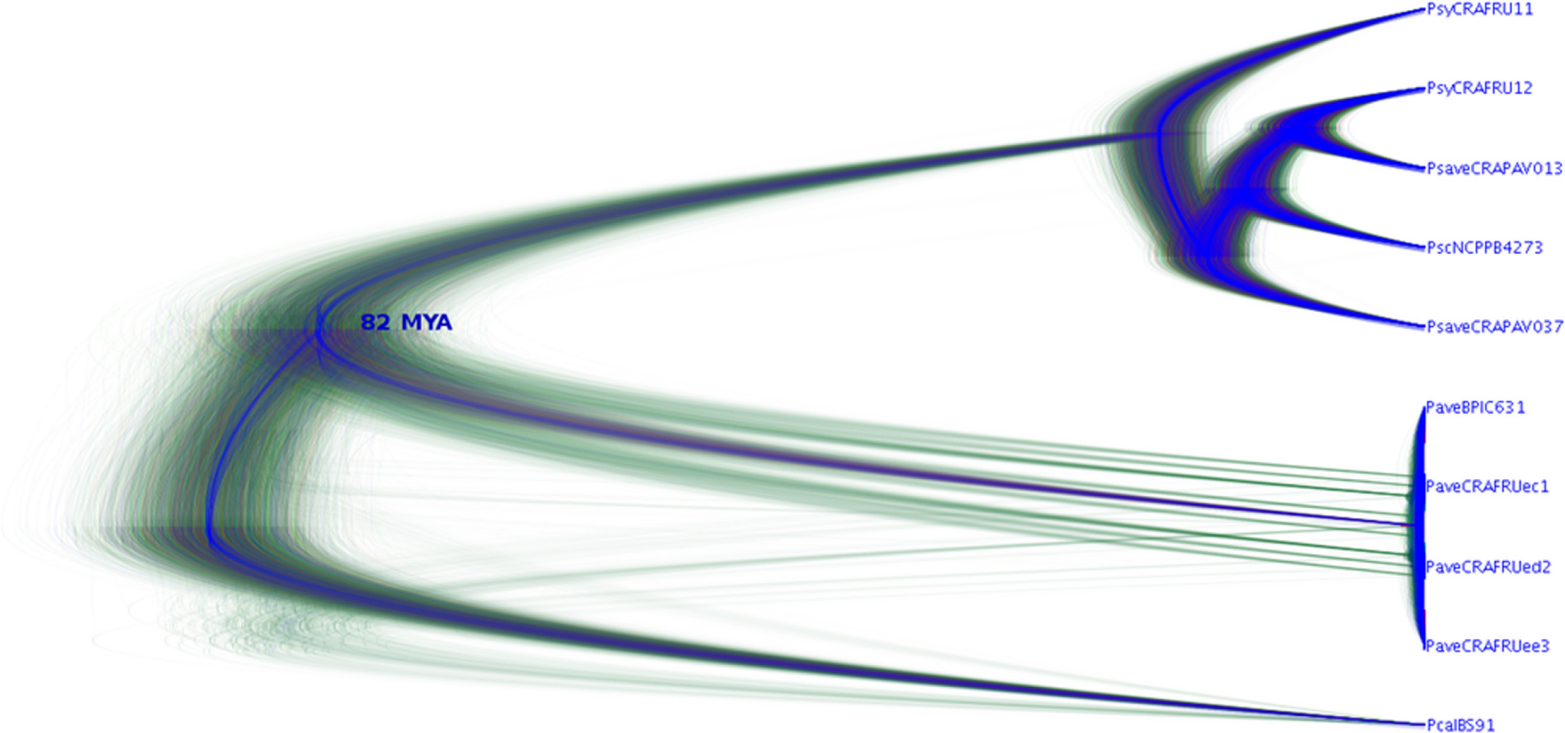
Divergence dendrogram based on the concatenated dataset of the four housekeeping genes. The dendrogram was built assessing *gapA*, *gltA*, *gyrB*, *rpoD* genes and by applying the *E*. *coli-Salmonella* model to nine pseudomonad strains infecting *Corylus avellana*. MYA: millions of years before present. Strain legend is shown in Table 1. *Pseudomonas cannabina* pv. *alisalensis* Pcal BS91 was included in the analysis as outgroup.

The putative divergence time between the two genomospecies for the four housekeeping genes was calculated according to two different estimations. By following the *Escherichia coli*-*Salmonella typhimurium* model based on a substitution rate of 1 x 10^9^ substitutions per year, the most common recent ancestor for the two species was estimated to have occurred between 41.8 and 142.0 million years ago. However, according to the *Staphylococcus aureus* model based on a substitution rate of 1 x 10^6^ substitutions per year, the most common recent ancestor for these two species instead occurred between 28.700 to 94.900 years before present (Table 4). The corresponding dendrogram based on the concatenated dataset of the four housekeeping genes obtained using the DensiTree software and built based on the *E*. *coli-S*. *typhimurium* model (Fig 4) suggests that the two clusters diverged approximately 82 millions of years before present.

**Table 4:** Divergence time estimates between *Pseudomonas avellanae* and *P*. *syringae* pathovars *avellanae*, *coryli* and *syringae* strains infecting *Corylus avellana* trees, according to the *Escherichia coli-Salmonella typhimurium* and *Staphylococcus aureus* models.

Calibration time	Rate (subst/yr)	Locus	Age of the most recent common ancestor in years (mean, 95% CI)
			*P*. *avellanae*–*P*. *syringae*
*E*. *coli–Salmonella*	1 X 10^9^	*gapA*	142.09 MY
		*gltA*	74.00 MY
		*gyrB*	89.76 MY
		*rpoD*	41.81 MY
*Staphylococcus aureus*	2 X 10^6^	*gapA*	94.900
		*gltA*	17.200
		*gyrB*	30.800
		*rpoD*	28.700

MY: millions of years before present.

### The type III secretion system effectors

**Fig 5:**
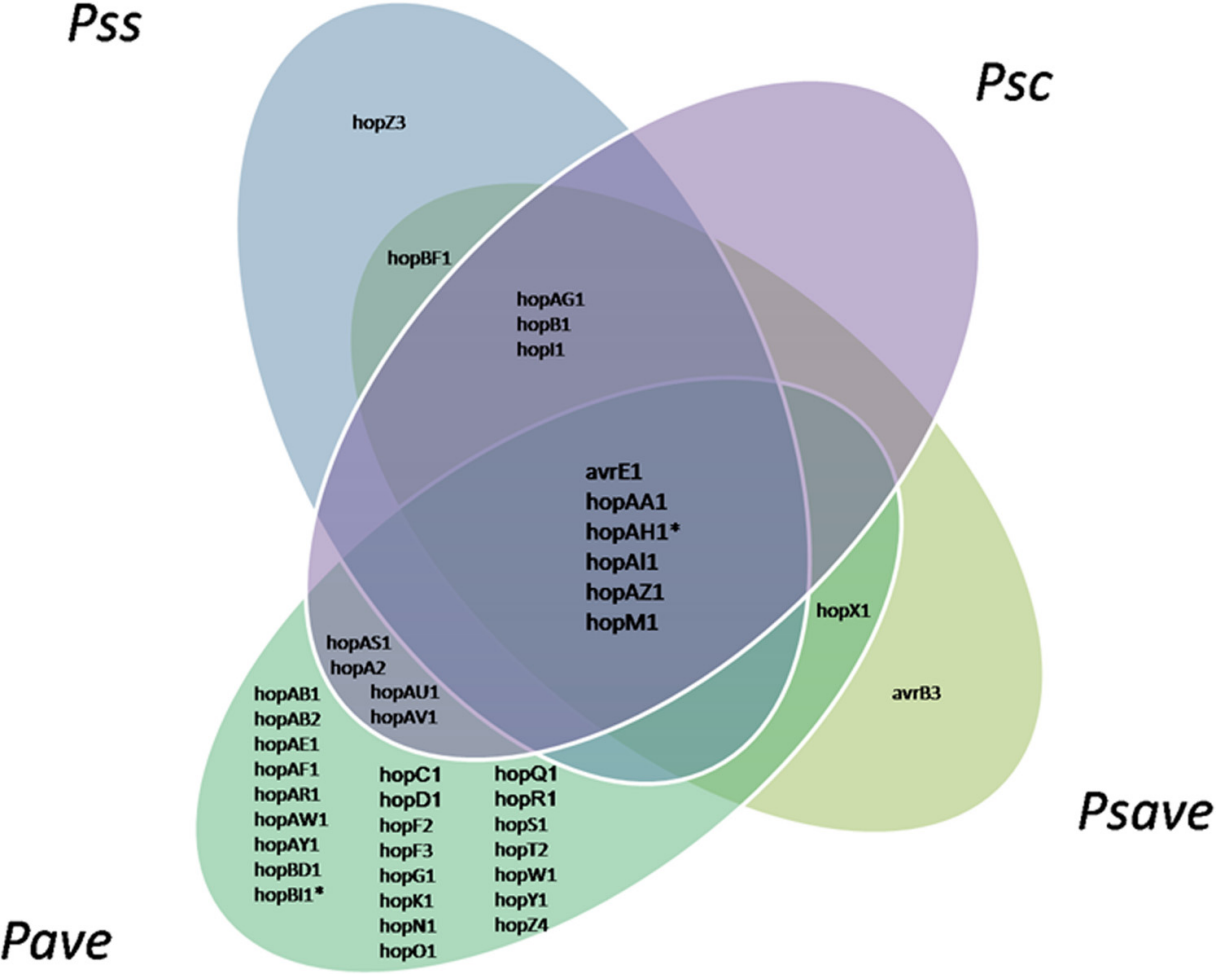
Venn diagram of the type III secretion system effector genes complement of nine pseudomonad strains infecting *Corylus avellana* trees. Pave: *Pseudomonas avellanae*; Psave: *P*. *syringae* pv. *avellanae*; Psc: *P*. *s*. pv. *coryli*; Pss: *P*. *s*. pv. *syringae*. *: not present in *P*. *avellanae* BPIC 631.

A comparison of the effector repertoires of the nine strains of phytopathogenic pseudomonads to infect *C*. *avellana* trees was completed based on the complete dataset of effector proteins found in the website database http://www.pseudomonas-syringae.org., e-10, length hit > 60%. The analysis revealed a core set of six putative effector genes that are conserved across all strains studied (Fig 5): *avrE1*, *hopAA1*, *hopAI1*, *hopAZ3*, *hopM1* and *hopAH1*, the latter of which was found to be absent in *P*. *avellanae* BPIC 631. In addition, *P*. *avellanae* and *P*. *syringae* pathovars *avellanae* and *syringae* showed a unique set of effector proteins with *hopZ3* and *avrB3* solely present in the *P*. *syringae* pathovars *syringae* and *avellanae*, respectively. Interestingly, *P*. *avellanae* had a significant higher number of effector proteins and unique effector protein genes. A comparison of the effector genes performed between the *P*. *avellanae* strains revealed that only BPIC 631 contained *hopBD1* (S4 Fig), whereas none of the three strains isolated in Italy possessed any unique effector gene. A comparison performed among the *P*. *syringae* pathovars strains infecting *C*. *avellana* showed that *P*. *s*. pv. *coryli* NCPPB 4273 and *P*. *s*. pv. *syringae* CRAFRU 11 showed distinct and unique repertoires of effector genes. Only *P*. *s*. pv. *coryli* had *hopA2*, *hopAS1*, *hopAU1* and *hopAV1*, while only *P*. *s*. pv. *syringae* CRAFRU 11 had *hopAX* and *hopZ3* (S5 Fig). The dendrogram of the relationships between the effector protein gene repertoires of the nine pseudomonads to infect *C*. *avellana* and obtained using UPGMA algorithm (S6 Fig) once more confirms the distinctiveness of the *P*. *avellanae* from the *P*. *syringae* pathovar strains.

### Genomic islands

**Fig 6:**
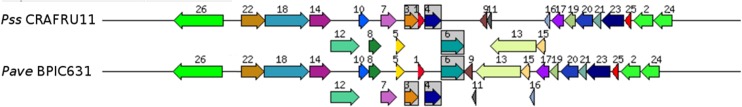
Structure of the genomic island Pcor GI1 of about 20 kb found in all nine pseudomonad strains infecting *Corylus avellana* trees. It was obtained using the SEED viewer comparative tool. The genomic structure of the island resulted conserved among the strains. Two representative structure regarding *Pseudomonas syringae* pv. *syringae* CRAFRU 11 and *P*. *avellanae* BPIC 631 are shown. Gene legend is shown in S2 Table.

The search for putative genomic islands within the genomes of the nine pseudomonads revealed the presence of Pcor GI1, a genomic island of approximately 20 kb present in all strains. The islands Pave GI2 and Pave GI3, of approximately 11.5 and 5 kbs, respectively, were only present in the *P*. *avellanae* strains. No variation in the sequences of these islands was found among the pseudomonads. Graphical representations of these genomic islands obtained with Seed Viewer (Fig 6) and R software (A, B, C in S7 Fig) show that Pcor GI1 contains many proteins of the *hrp/hrc* type III secretion system cluster. These include HrpK1, a member of a conserved family of the type III secretion system translocon components, and Hrc QA and Hrc QB, conserved component of the bacterial type III secretion system (Table 5 and S2 Table). Of the proteins found only in the *P*. *avellanae* strains, Pave GI2 contains a methyl-accepting chemotaxis protein, whereas Pave GI3 contains proteins related to the prophage PSPPH04.

**Table 5:** Gene content in the region of the three genomic islands found in *Pseudomonas avellanae* and *P*. *syringae* pathovars *avellanae*, *coryli* and *syringae* strains all infecting *Corylus avellana* trees.

Genomic Island	Accession number	Protein
Pcor GI1		tRNA-Leu
	NP_791232	type III helper protein HrpK1
	ZP_03396399	RNA polymerase sigma factor HrpL
	NP_791230	type III secretion protein HrpJ
	NP_791229	type III secretion protein HrcV
	NP_791228	type III secretion protein HrpQ
	NP_791227	type III secretion cytoplasmic ATPase HrcN
	ZP_03396404	type III secretion protein HrpO
	NP_791225	type III secretion protein HrpP
	ZP_03396406	type III secretion protein HrcQA
	ZP_03396407	type III secretion protein HrcQB
	NP_791222	type III secretion protein HrcR
	NP_791221	type III secretion protein HrcS
	NP_791220	type III secretion protein HrcT
	NP_791219	type III secretion protein HrcU
	NP_791216	outer-membrane type III secretion protein HrcC
	NP_791218	negative regulator of hrp expression HrpV
	NP_791215	type III secretion protein HrpG
	NP_791214	type III secretion protein HrpF
	NP_791213	type III secretion protein HrpE
	ZP_03396418	type III secretion protein HrpD
	NP_791211	type III secretion protein HrcJ
	NP_791210	type III secretion protein HrpB
	NP_791209	type III helper protein HrpZ1
	ZP_03396422	type III helper HrpA1
	ZP_03396423	type III transcriptional regulator HrpS
	ZP_03396424	type III transcriptional regulator HrpR
Pave GI2		tRNA-Met
	ZP_07003726	Integrase
	YP_235890	hypothetical protein Psyr_2813
	ZP_06459683	hypothetical protein PsyrpaN_16564
	YP_672807	hypothetical protein Meso_0238
	ZP_07003719	hypothetical protein PSA3335_1054
	ZP_04625031	TonB-dependent siderophore receptor
	YP_001669551	anti-FecI sigma factor
	YP_001669552	ECF subfamily RNA polymerase sigma-24 factor
	ZP_07004897	Methyl-accepting chemotaxis protein
Pave GI3		tRNA-Leu
	ZP_06496267	tail fiber domain-containing protein
	ZP_06496269	tail fiber assembly domain-containing protein
	YP_274596	prophage PSPPH04
	YP_001347798	hypothetical protein PSPA7_2431
	NP_793797	hypothetical protein PSPTO_4035

Pcor GI 1 was found present in all nine strains, whereas Pave GI 2 and Pave GI 3 were revealed solely in the *P. avellanae* strains.

### Presence of phytotoxins and antimetabolites

**Fig 7:**
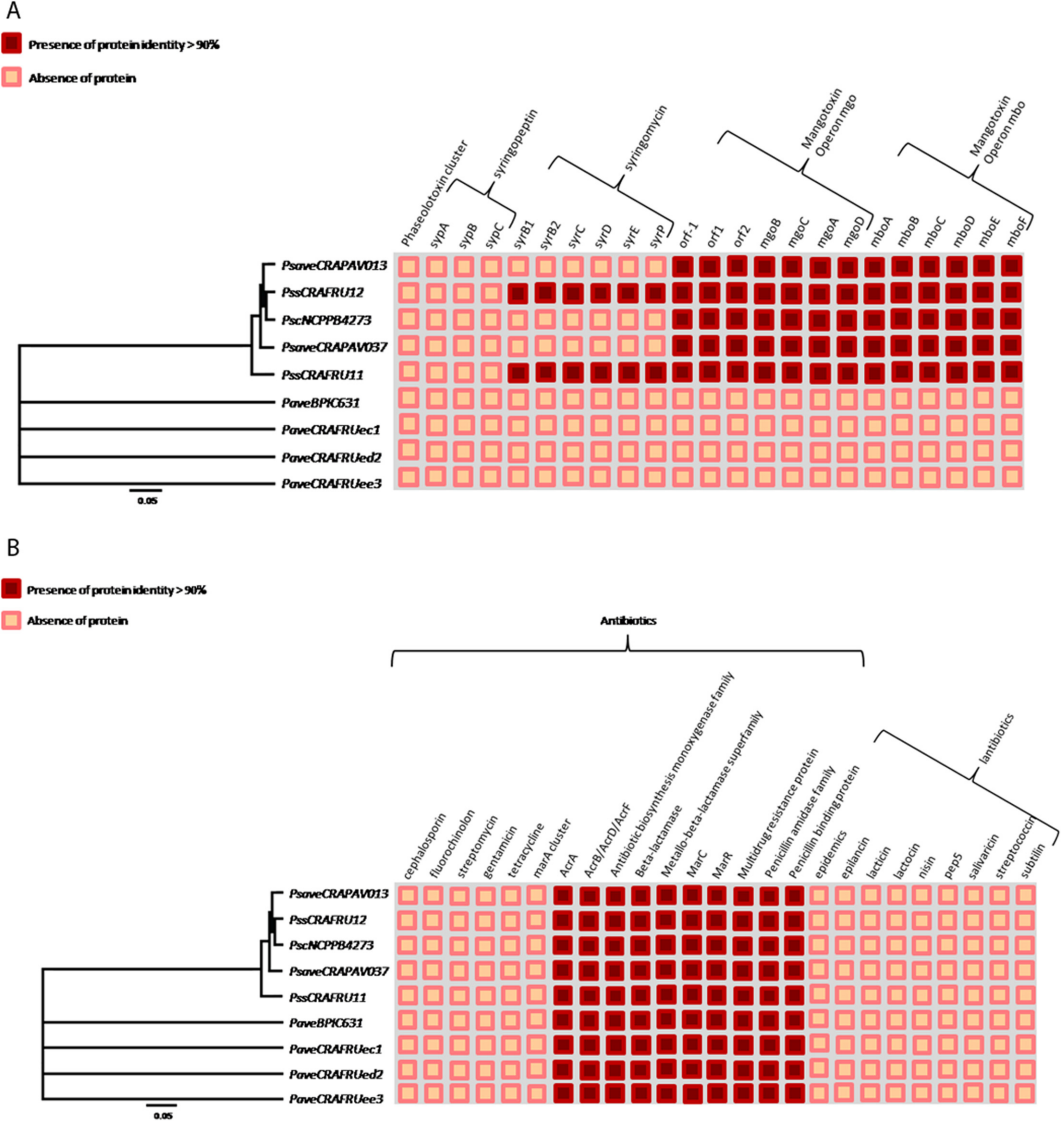
Presence/absence of putative homolog proteins in nine pseudomonad strains infecting *Corylus avellana* trees. The indication concerns the production of phytotoxins and antimetabolites (A), and antibiotic and lantibiotics (B). Strain legend is shown in Table 1.

The assessment for the presence of homologues to phytotoxins and antimetabolites revealed a remarkable difference between *P*. *avellanae* and the *P*. *syringae* pathovars. In fact, the four *P*. *avellanae* strains did not display any homolog protein related to phaseolotoxin, syringopeptin, syringomycin or mangotoxin. In contrast, *P*. *s*. pathovars *avellanae*, *coryli*, and *syringae* all contain the entire protein cluster of the mangotoxin operons Mgo (MgoBCAD) and Mbo (MboABCDEF). In addition, the two *P*. *s*. pv. *syringae* strains were found to possess the syringomycin cluster, that is not present in *P*. *s*. pathovars *avellanae* and *coryli*. Neither phaseolotoxin nor syringopeptin was identified in any *P*. *syringae* strains (Fig 7A).

### Antibiotic and lantibiotic detoxification, bacteriocins and copper resistance

**Fig 8:**
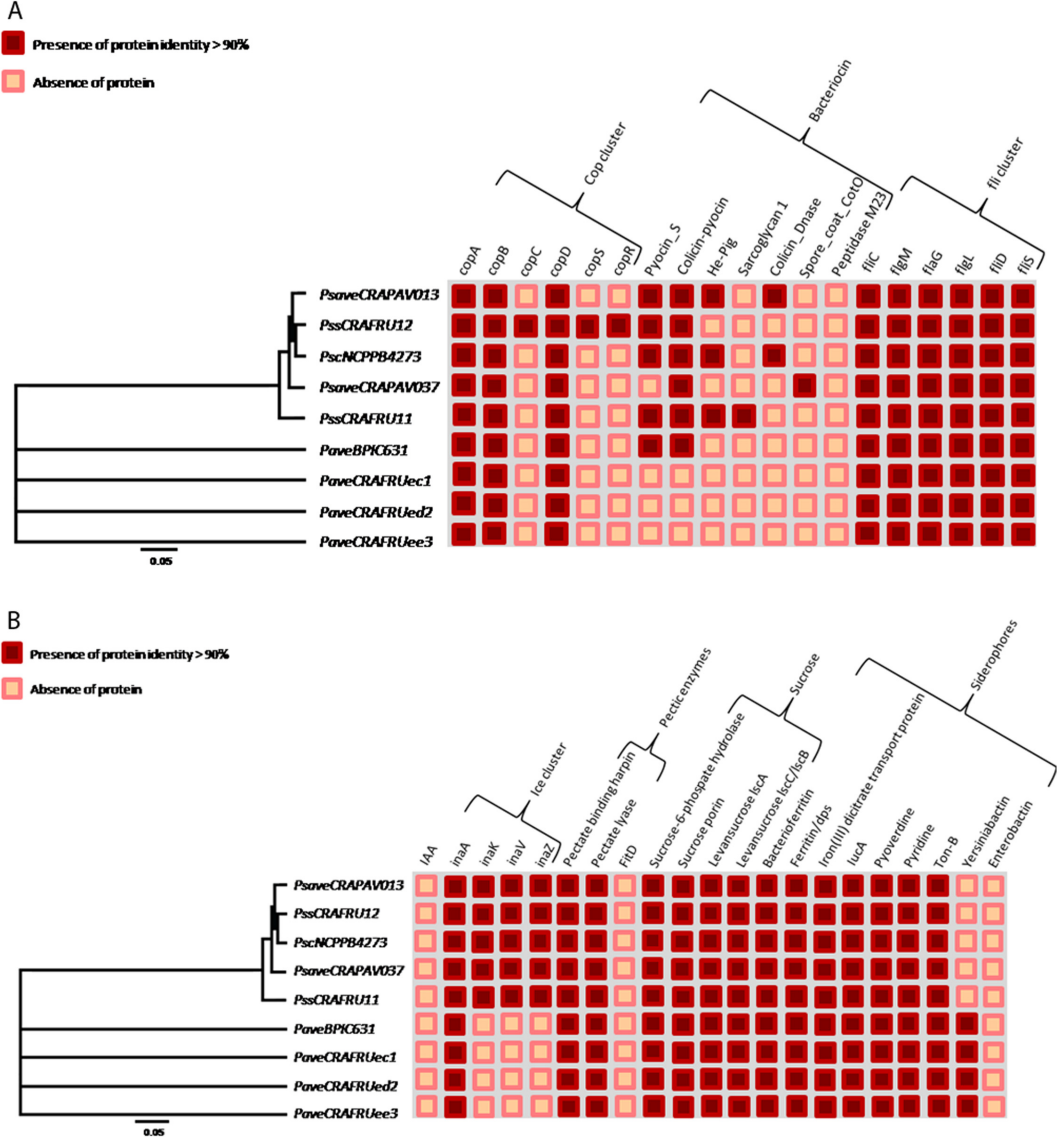
Presence/absence of putative homolog proteins in nine pseudomonad strains infecting *Corylus avellana* trees. The indication concerns the presence of copper resistance genes, bacteriocins, and the flagellin cluster (A), and ice nucleation activity, pectic enzymes, sucrose metabolism, and siderophores (B). Strain legend is shown in Table 1.

No homologues proteins related to lantibiotic detoxification were found in any strain. By contrast, the nine pseudomonad strains contained an array of homologs involved in the detoxification of antibiotics such as multidrug resistance protein, beta-lactamase, penicillin, MarC and MarR (Fig 7B). Among the bacteriocins, the pyocins were found to be the most broadly distributed, especially within the *P*. *syringae* pathovars. However, the three *P*. *avellanae* strains isolated in Italy did not possess any homolog related to bacteriocin production (Fig 8A). Some homologues proteins of the Cop operon related to copper resistance were found in all of the nine pseudomonads infecting *C*. *avellana* trees. In particular, CopA, CopB and CopD were present in all nine strains. Of note, only *P*. *s*. pv. *syringae* CRAFRU 12 contained all of the homologues within this cluster (Fig 8A), including CopC, CopR and CopS. (Fig 8A). All nine pseudomonads contain the same set of the *fli* cluster genes coding for flagellin.

### Siderophores, pectic enzymes, sucrose metabolism, and ice nucleation activity

All nine strains displayed a vast number of siderophores, including bacterioferrin, ferritin, pyoverdine, pyridine, and the Ton-B iron transporter. By contrast, none of the strains were found to possess enterobactin. Of note, only the *P*. *avellanae* strains contained yersiniabactin (Fig 8B). All nine strains were found to have pectin enzymes and the proteins related to sucrose metabolism such as sucrose-6-phosphate hydrolase, sucrose porin and levansucrase. Conversely, none of the nine strains were found to have homologs to indolacetic acid or the FitD anti-insect toxin. Only the five *P*. *syringae* pathovar strains contained the entire ice nucleation activity cluster (Fig 8B).

### Type IV and type VI secretion systems, flagellin and CRISPRs

The nine pseudomonad strains infecting the European hazelnut display most of the homologues to type IV secretion system Pil cluster of pilin. In addition, all nine strains possess homologues of the bacterial flagellin and of the type VI secretion system cluster, although in the *P*. *avellanae* strains was found solely the Vgr family protein homologues, these genes remarkably not present in the *P*. *syringae* strains (S8 Fig). CRISPRs were not found in any of the nine strains.

## Discussion

This study demonstrated that strains of *P*. *avellanae* and *P*. *syringae* pathovars *avellanae*, *coryli* and *syringae* infecting *C*. *avellana* trees belong to two different genomic clusters. Any approaches used here yielded clearly separated the clusters, including the MLSA and neighbor-net performed with four housekeeping genes, the consensus network and neighbor net performed with 2.812 trees and 1.977.984 nucleotides, respectively, or the reciprocal identity of the draft genomes of lower than 95%, assessed by ANI/TETRA analyses. These results confirmed and expanded on the previous taxonomic studies on plant pathogenic pseudomonads that had also been performed with DNA-DNA hybridization, molecular typing and draft genome comparisons on members of the *P*. *syringae* complex. These previous studies reliably separated these pseudomonads into nine discrete genomospecies, with *P*. *avellanae* as genomospecies 8 and *P*. *syringae* pathovars *avellanae*, *coryli* and *syringae* as genomospecies 1 [6, 8, 9, 22].

Our data are also strongly supported by the recent study of Nowell *et al*. [52] that placed strains of *P*. *s*. pv. *syringae* (i.e., phylogroup 2) in a distinct cluster separated from strains of *P*. *s*. pv. *actinidiae* and *P*. *s*. pv. *morsprunorum* (i.e., phylogroup 1), two pathovars belonging to *P*. *avellanae* species as previously shown [8, 9]. Based on the evolutionary relationships among 27 pathovars of the *P*. *syringae* complex and inferred from 1 million orthologous nucleotide sites of the core genomes, Nowell *et al*. [52] stated that the phylogroups diverged from each other as separate species or genera. In addition, they identified no significant recombination event between the core alleles of the phylogroups that could erode their distinctiveness. Similarly, the present study demonstrated the absence of recombination between the two *Pseudomonas* clusters infecting the European hazelnut. A series of analyses, including the recombination network performed with the four housekeeping genes, the consensus network performed with 2.812 trees, the neighbour-net analysis and the likelihood-based model selection procedure, did not reveal any recombination event between the genes of the tested strains. Consistent with these findings, there were also high F_ST_ index values estimating the gene flow between the two groups, suggesting an absence in gene shuffling between them.

ClonalFrame analysis provided further confirmation of the distinctiveness of the pseudomonads causing disease to *C*. *avellana*. This method infers the clonal relationship of bacterial strains by accounting for both recombination and point mutation events; it has also been used to reveal putative common ancestors [43]. ClonalFrame analysis of the MLSA genes (*gapA*, *gltA*, *gyrB* and *rpoD*) supported the distinctiveness of these two groups. In addition, the coalescent tree indicated that the *P*. *syringae* pathovar strains had a more recent common ancestor than *P*. *avellanae*. However, no recent common ancestor was found for the two groups. Finally, the McDonald-Kreitman tests on the four housekeeping genes of the two clusters to assess for adaptive divergence did not show any evidence of positive selection. Collectively, these results suggest that two distinct *Pseudomonas* belonging to two different genetic clusters converged onto *C*. *avellana* to display their pathogenic behaviour. These data combined with the study of Nowell *et al*. [52], strongly support the hypothesis that the *P*. *syringae* complex might be also considered to be composed of discrete species with cases of overlapping host range due to the possible pathogenic convergent evolution to the same host plant.

Depending on the substitution rate used there could be large variation between the estimated divergence time calculation. According to the *E*. *coli*-*S*. *typhimurium* model, the separation of the two clusters took place between approximately 42 and 142 million of years ago, while according to a lower substitution rate model based on *Staphylococcus aureus*, the two groups diverged between approximately 29.000 and 95.000 years ago. Both models can explain the putative association between the two groups as colonizers of the *Betulaceae* family to which *C*. *avellana* belongs [53].

This study also identified a genomic island of approximately 20 kb in the nine pseudomonads infecting European hazelnut trees. This island contains the *hrp/hrp* cluster which codes for the type III secretion system. This system is a fundamental apparatus for injecting pathogenicity effector proteins. The acquisition of genomic island(s), possibly by horizontal gene transfer, can play a major role in remodelling the genomic structure and altering the complement of virulence factors [54]. Like other plant pathogenic pseudomonads [55], also for *P*. *avellanae* and the *P*. *syringae* pathovars *avellanae*, *coryli* and *syringae*, all infecting *C*. *avellana*, a genomic island might have played a fundamental role in allowing these pathogens to occupy a new ecological niche (i.e., a new host plant) and/or to enlarge the host plant range, as is the case for the pathovar *syringae*.

This study identified a significantly higher number of type III secretion system effector proteins in the *P*. *avellanae* strains than in the *P*. *syringae* strains. A relatively low number of type III secretion system effectors was already found in other *P*. *s*. pv. *syringae* strains such as FF5 which was isolated from *Pyrus calleryana* [56]. We also noted different repertoires of effectors between the two species. This finding confirms the results of O’Brien *et al*. [31] and the extensive effector remodelling found in the strains assessed in such a study could be also explained by the fact that those strains belong to two different genetic clusters. The *P*. *syringae* pathovar strains studied here all contained the operons Mbo and Mgo for the production of mangotoxin production, an antimetabolite toxin playing a major role in the *P*. *s*. pv. *syringae* strains causing apical necrosis of mango trees [57]. This feature was not found in the *P*. *avellanae* strains. By assessing 94 strains belonging to six genomospecies *sensu* Gardan *et al*. [6], Carrión *et al*. [58] found that the Mbo operon was consistently present in the *P*. *syringae* strains of genomospecies 1, namely in pathovars *aptata*, *avellanae*, *japonica*, *pisi* and *syringae*. This operon is retained essential for the biosynthesis of mangototoxin [59]. We confirmed its presence in *P*. *s*. pv. *avellanae* and in two other *P*. *s*. pv. *syringae* strains isolated from *C*. *avellana* and identifed the operon in another *P*. *syringae* pathovar, namely *coryli*, belonging to genomospecies 1 [22]. Of note, this operon was highly conserved in the different *P*. *syringae* pathovar strains. suggesting that its acquisition occurred by lateral gene transfer [59]. Mangotoxin production is regulated by MgoA, a nonribosomal peptide synthetase in the Mgo operon [60]. The presence of MgoA homologs was found in all *P*. *syringae* pathovars causing disease in the European hazelnut. Intriguingly, despite the fact that none of the *P*. *avellanae* strains possessed any phytotoxin or antimetabolites, this pathogen was more devastating to *C*. *avellanae* than the *P*. *syringae* pathovars. This finding confirmed the fact that phytotoxins and antimetabolites virulence factors are not always responsible for major damages to host plants.

The nine pseudomonads infecting the European hazelnut did not show differences concerning in the set of homologues proteins related to antibiotic/lantibiotic detoxification. Apparently, these strains are not able to counteract lantibiotics. Lantibiotics are peptide antibiotics with one or more thioether bonds [61]; they are produced by Gram-positive bacteria such as *Streptomyces* and *Streptococcus* and show a strong antimicrobial activity towards Gram-negative bacteria [62]. By contrast, the nine pseudomonad strains were found to possess hortologs that may act to confer protection or resistance to several common antibiotics, such as beta-lactamase, penicillin and resistance to multiple antibiotics. This putative resistance may provide them with the possibility to compete *in planta* with other bacteria and fungi.

Bacteriocins are antimicrobial peptides with a narrower range of activity than antibiotics; however, they are more effective against sensitive microbes [63]. While the three *P*. *avellanae* strains isolated in Italy do not appear to contain any protein related to bacteriocin compounds, all the other strains putatively produce some bacteriocins. These bacteriocins include the pyocins, that were first identified in *P*. *aeruginosa* and are involved in the suppression of closely related microbes [64]. Similarly, putative homologues for pyocin have been found to be present in other *P*. *s*. pv. *syringae* strains (B728a) and *P*. *syringae* pathovars (*P*. *s*. pv. *phaseolicola* 1448A) [65, 66]. Whether this enhances the environment fitness of the phytopathogenic bacteria remains to be determined.

The full set of copper-resistance homologue proteins conferring resistance to copper has been found only in one *P*. *s*. pv. *syringae* strain, namely CRAFRU 12, even though copA, copB and copD were found to be present in all strains. Interestingly, the putative homologue of the Cop operon found in CRAFRU 12, namely CopABCDRS, was previously found and characterized in plasmid pPT23D of the *P*. *s*. pv. *tomato* strains in the U.S.A. [67, 68]. A highly similar Cop operon was also found in many *P*. *s*. pv. *actinidiae* strains isolated from *Actinidia deliciosa* in Japan [69]. Whether this operon was acquired by *P*. *s*. pv. *syringae* CRAFRU 12 through lateral gene transfer from another phytopathogenic or a saprophyte bacterium [70] is not known; however, this question merits further research attention.

A vast array of siderophores, including bacterioferrin, ferritin, pyoverdine, pyridine, and the ton-B iron transporter, were found to be present in the nine pseudomonad strains infecting *C*. *avellana*. The siderophores are likely to confer good *in planta* fitness and competiveness. Of note, only the *P*. *avellanae* strains possess putative homologues to yiersiniabactin, a siderophore with a very high stability constant for iron. This result confirm and expands the work of Bultreys *et al*. [71], who used PCR to detect homologues to yiersiniabactin in *P*. *s*. pv. *theae* LMG 5092, another strain of genomospecies 8 [9], but no homologues in the *P*. *syringae* strains of genomospecies 1, including the pathotype strain of the pathovar *syringae*. This siderophore also plays a relevant role in the field control of bacterial phytopathogens through copper compounds. In fact, it has been shown that yersiniabactin binds copper and prevents its cathecol-mediated reduction, thus counteracting its toxic effect to the bacterial cell [72].

Of the pectic enzymes, putative homologs of pectate lyase, an effective cell wall-degrading enzyme, have been found to be present in all nine strains. This enzyme has been previously found in other *P*. *syringae* pathovars, including *glycinea*, *lachrymans*, *phaseolicola*, *tabaci* and *tagetis* [73, 74]. Concerning the phytopathogenic pseudomonads, this enzyme is involved in the final steps of plant tissue maceration. However, it is not involved in the pathogenicity and/or shifting of the host range [73]. All nine strains displayed homologues to levansucrase, LscA and LscB/C [75], an enzyme that utilizes sucrose to produce glucose and levan, the latter of which is an exopolysaccharide involved in the pathogenicity of many plant pathogenic bacteria [76]. All strains contained homologues of the bacterial flagellin cluster, including FliS which codes for a specific chaperone of the flagellum [77]. Flagellin is a well-known elicitor of the microbe-associated molecular pattern (MAMP) molecules [78]. By contrast, these strains do not contain homologues to indolacetic acid production and to the anti-insect activity toxin Fit found in *P*. *fluorescens* [79]. Homologues of the type VI secretion system [80] have been found in the strains assessed here, even though in the *P*. *avellanae* strains only the Vgr family protein was revealed, such a family protein was not found in the *P*. *syringae* pathovar strains here assessed. In addition, homologues of the type IV secretion system cluster were found to be present in these strains. This secretion system codes for pilin, an adhesion involved in the bacterial twitching motility. This adhesin allows the pathogen to explore surfaces and form a biofilm [81, 82], thus fundamentally affecting its pathogenicity.

Taken together, these results revealed that strains belonging to two different *Pseudomonas* genetic clusters can cause disease in the same host plant, *C*. *avellana*. These pathosystems therefore represent an outstanding case of pathogenic convergence.

By exploiting different virulence factors, both *P*. *avellanae* and *P*. *syringae* pathovars *avellanae*, *coryli* and *syringae* incite twig and branch wilting, while *P*. *avellanae* and *P*. *s*. pv. *avellanae* also cause tree death. The possible horizontal transfer of a genomic island containing the *hrp/hrc* cluster coding for the type III secretion system could have played a fundamental role in partially modifying their lifestyle(s). In fact, the en bloc transfer of genetic elements involved in the pathogenicity of microbes can induce dramatic changes in the behaviour of the recipient strain [83]. The evolution of locally adaptive gene clusters conferring new genetic trait(s) to the recipient host is currently being investigated in a broader genomic and ecological context [84]. Within this context, it has been shown that genes whose products interact directly with rapidly changing biotic or abiotic environments such as those that function in disease resistance mechanisms, had a higher probability of transposition in *Arabidopsis* [85]. The possibility of a high rate of transmission of genomic island(s) between phytopathogenic bacteria, thus conferring new adaptations to the recipient cells is an issue that merits further in-depth studies. Some distinctive features of the strains of the two genetic clusters assessed here might also be related to differences in their *in planta* fitness and/or in their mechanism(s) of host colonization. In fact, the *P*. *syringae* pathovars contain homologues to ice nucleation activity and to mangotoxin production, while the *P*. *avellanae* strains possess homologues to the potent siderophore yersiniabactin. The precise role of these molecules is still to assess in these phytopathogens. However, based on information from other plant pathogenic bacteria their role might be relevant for *Pseudomonas* infection of *C*. *avellana*. Finally, to more precisely understand the ecology of plant pathogenic bacteria, beyond simply knowing which plant host they colonize, future research should focus on identifying other possible habitats that these species might occupy (i.e., water, soil, wild flora and animals, insects).

## Supporting Information

S1 Fig Field symptoms induced by *Pseudomonas avellanae* and *P*. *syringae* pathovars *avellanae*, *coryli* and *syringae* to *Corylus avellana* trees. A) tree death caused by *P*. *avellanae*; B) longitudinal canker on trunk incited *P*. *avellanae*; C) tree death caused by *P*. *syringae* pv. *avellanae*; D) twig wilting caused by *P*. *s*. pv. *coryli*; E) branch die-back incited by *P*. *s*. pv. *syringae*.
(TIF)

S2 Fig Neighbor-net tree based on the concatenated sequences of four housekeeping genes. Tree based on split analysis of *gapA*, *gltA*, *gyrB* and *rpoD*, for a total of 6.812 nucleotides of *Pseudomonas avellanae* and *P*. *syringae* pathovars *avellanae*, *coryli* and *syringae* infecting *Corylus avellana* trees. Bootstrap values are shown at the main nodes. The scale bar indicates the number of substitutions per nucleotide position. Strain legend is shown in Table 1. *P*. *cannabina* pv. *alisalensis* Pcal BS91 was included in the analysis as outgroup.
(TIF)

S3 Fig Coalescent tree showing the clonal genealogy of nine pseudomonad strains infecting *Corylus avellana*. The tree is based on the partial sequence of four housekeeping genes (i.e., *gapA*, *gltA*, *gyrB*, *rpoD*). Strain legend is shown in Table 1.
(TIF)

S4 Fig Venn diagram of the type III secretion system effector gene complement of *Pseudomonas avellanae* strains isolated in Greece and Italy. Strain legend is shown in Table 1.
(TIF)

S5 Fig Venn diagram of the type III secretion system effector gene complement of *Pseudomonas syringae* pathovars avellanae, coryli and syringae strains. Strain legend is shown in Table 1.
(TIF)

S6 Fig Relationships among the type III secretion systems effector gene complement of nine pseudomonad strains infecting *Corylus avellana*. The dendrogram is based on the presence/absence of the effectors obtained using UPGMA algorithm. Strain legend is shown in Table 1. *Pseudomonas cannabina* pv. *alisalensis* Pcal BS91 was included in the analysis as outgroup.
(TIF)

S7 Fig Graphical representation of the three genomic islands found in the nine pseudomonad strains infecting *Corylus avellana* trees. It was obtained using R software. Arrows indicate the beginning and the end of the island. Putative hortolog genes are indicated. A) Pcor GI 1, identified in all pseudomonad strains infecting *C*. *avellana*; B) and C) Pave GI2 and Pave GI3, respectively, identified solely in the *Pseudomonas avellanae* strains.
(TIF)

S8 Fig Presence/absence of putative homolog proteins in nine pseudomonad strains infecting *Corylus avellana* trees. The indication concerns the presence of type IV and type VI secretion systems homologues. Strain legend is shown in Table 1.
(TIF)

S1 Table Type of symptom induced by different phytopathogenic pseudomonads to *Corylus avellana* trees and its relative severity.-: absence of symptom. (DOC)

S2 Table Strain content legend of the genomic island Pcor GI 1 found present in all pseudomonad strains infecting *Corylus avellana* trees. (DOC)

## References

[1] Psallidas PG. The problem of bacterial canker of hazelnut in Greece caused by *Pseudomonas syringae* pv. *avellanae* . EPPO Bull. 1987; 17: 257–261.

[2] Scortichini M. Bacterial canker and decline of European hazelnut. Plant Disease. 2002; 86: 704–709. 10.1094/PDIS.2002.86.7.704 30818564

[3] Psallidas PG. *Pseudomonas syringae* pv. *avellanae*, pathovar nov., the bacterium causing canker disease on *Corylus avellana* . Plant Pathol. 1993; 42: 358–363.

[4] Scortichini M, Tropiano FG. Severe outbreak of *Pseudomonas syringae* pv. *avellanae* on hazelnut in Italy . J Phytopathol. 1994; 140: 65–70.

[5] Janse JD, Rossi MP, Angelucci L, Scortichini M, Derks JHJ, Akkermans ADL, et al Reclassification of *Pseudomonas syringae* pv. *avellanae* as *Pseudomonas avellanae* (spec. nov.), the bacterium causing canker of hazelnut (*Corylus avellana* L.). Syst Appl Microbiol. 1996; 19, 589–595.

[6] Gardan L, Shafik H, Belouin S, Broch R, Grimont F, Grimont PA. DNA relatedness among the pathovars of *Pseudomonas syringae* and description of *Pseudomonas tremae* sp. nov. and *Pseudomonas cannabina* sp. nov. (*ex* Sutic and Dowson 1959). Int J Syst Bacteriol. 1999; 49: 469–478. 10319466 10.1099/00207713-49-2-469

[7] Sarkar SF, Guttman DS. Evolution of the core genome of *Pseudomonas syringae*, a highly clonal, endemic pathogen. Appl Environ Microbiol. 2004; 70; 1999–2012. 15066790 10.1128/AEM.70.4.1999-2012.2004 PMC383139

[8] Scortichini M, Marcelletti S, Ferrante P, Firrao G. A genomic redefinition of *Pseudomonas avellanae* species. PLoS ONE. 2013; 8: e75794 10.1371/journal.pone.0075794 24086635 PMC3783423

[9] Marcelletti S, Scortichini M. Definition of plant-pathogenic *Pseudomonas* genomospecies of the *P*. *syringae* complex through multiple comparative approaches. Phytopathology. 2014; 104: 1274–1282. 10.1094/PHYTO-12-13-0344-R 24875383

[10] Berge O, Monteil CL, Bartoli C, Chandeysson C, Guilbaud C, Sands DC, et al A user’s guide to a data base of the diversity of *Pseudomonas syringae* and its application to classifying strains in this phylogenetic complex. PLoS ONE. 2014; 9: e105547 10.1371/journal.pone.0105547 25184292 PMC4153583

[11] Scortichini M, Natalini E, Marchesi U. Evidence for separate origins of the two *Pseudomonas avellanae* lineages. Plant Pathol. 2006; 55: 451–457.

[12] Wang PW, Morgan RL, Scortichini M, Guttman DS. Convergent evolution of phytopathogenic pseudomonads onto hazelnut. Microbiol. 2007; 153: 2067–2073. 10.1099/mic.0.2006/001545-0 17600051

[13] Scortichini M, Dettori MT, Rossi MP, Marchesi U, Palombi MA. Differentiation of *Pseudomonas avellanae* strains from Greece and Italy by rep-PCR genomic fingerprinting. J Phytopathol. 1998; 146: 417–420.

[14] Scortichini M, Marchesi U, Angelucci L, Rossi MP, Dettori MT. Occurrence of *Pseudomonas avellanae* (Psallidas) Janse *et al*. and related pseudomonads on wild *Corylus avellana* trees and genetic relationships with strains isolated from cultivated hazelnut. J Phytopathol. 2000; 148: 523–532.

[15] Natalini E, Scortichini M. Variability of 16S-23S rRNA gene internal transcribed spacer in *Pseudomonas avellanae* strains. FEMS Microbiol Lett. 2007; 271: 274–280. 17442015 10.1111/j.1574-6968.2007.00725.x

[16] Kaluzna M, Ferrante P, Sobiczewski P, Scortichini M. Characterization and genetic diversity of *Pseudomonas syringae* from stone fruits and hazelnut using repetitive-PCR and MLST. J Plant Pathol. 2010; 92: 781–787.

[17] Scortichini M, Rossi MP, Loreti S, Bosco A, Fiori M, Jackson RW, et al (2005) *Pseudomonas syringae* pv. *coryli* (pv. nov.), the causal agent of bacterial twig dieback of *Corylus avellana* L. Phytopathology. 2005; 95: 1316–1324. 10.1094/PHYTO-95-1316 18943363

[18] Cirvilleri G, Scuderi G., Bonaccorsi A, Scortichini M. Occurrence of *Pseudomonas syringae* pv. *coryli* on hazelnut orchards in Sicily, Italy and characterization by fluorescent amplified fragment length polymorphism. J Phytopathol. 2007; 155: 397–402.

[19] Poschenrieder G, Czech I, Friedrich-Zorn M, Huber B, Theil S, Janse JD, et al Erster nachweiss von *Pseudomonas syringae* pv. *coryli* (pv. nov.) und *Xanthomonas arboricola* pv. *corylina* an *Corylus avellana* (Haselnuss) in Deutschland. Bayerische Landesanstalt für Landwirtschaft-Insitut fur Pflanzenschutz. Jahresbericht 2005 2006; 32–33.

[20] Loreti S, Sarrocco S, Gallelli A. Preliminary investigation of *hrpZ* gene presence in *Pseudomonas avellanae* and in a bacterium inducing HR on tobacco. J Plant Pathol. 1999; 81: 234.

[21] Scortichini M, Marchesi U, Rossi MP, Di Prospero P. Bacteria associated with hazelnut (*Corylus avellana* L.) decline are of two groups: *Pseudomonas avellanae* and strains resembling *P*. *syringae* pv. syringae. Appl Environ Microbiol. 2002; 68: 476–484. 11823181 10.1128/AEM.68.2.476-484.2002 PMC126672

[22] Loreti S, Gervasi F, Gallelli A, Scortichini M. Further molecular characterization of *Pseudomonas syringae* pv. *coryli* . J Plant Pathol. 2008; 90: 57–64.

[23] Scortichini M, Lazzari M. Systemic migration of *Pseudomonas syringae* pv. *avellanae* in twigs and young trees of hazelnut and symptom development. J Phytopathol.1996; 144: 215–219.

[24] Scortichini M, Loreti S. Occurrence of an endophytic, potentially pathogenic strain of *Pseudomonas syringae* in symptomless wild trees of *Corylus avellana* L. J Plant Pathol. 2007; 89: 431–434.

[25] Almeida NF, Yan S, Lindeberg M, Studholme DJ, Schneider DJ Condon B, et al A draft genome sequences of *Pseudomonas syringae* pv. *tomato* T1 reveals a type III effector repertoire significantly divergent from that of *Pseudomonas syringae* pv. *tomato* DC3000. Mol Plant Microbe Interact. 2009; 22: 52–62. 10.1094/MPMI-22-1-0052 19061402

[26] Baltrus DA, Nishimura MT, Romanchuk A, Chang JH, Mukhtar MS, Cherkis K, et al Dynamic evolution of pathogenicity revealed by sequencing and comparative genomics of 19 *Pseudomonas syringae* isolates. PLoS Pathog. 2011; 7: e1002132 10.1371/journal.ppat.1002132 21799664 PMC3136466

[27] Marcelletti S. Ferrante P, Petriccione M, Firrao G, Scortichini M. *Pseudomonas syringae* pv. *actinidiae* draft genomes comparison reveal strain-specific features involved in adaptation and virulence to *Actinidia* species. PLoS ONE. 2011; 6: e27297 10.1371/journal.pone.0027297 22132095 PMC3223175

[28] Mc Cann HC, Rikkerink EH, Bertels F, Fiers M, Lu A, Rees-George J, et al Genomic analysis of the kiwifruit pathogen *Pseudomonas syringae* pv. *actinidiae* provides insight into the origins of an emergent plant disease. PLoS Pathog. 2013; 9: e1003503 10.1371/journal.ppat.1003503 23935484 PMC3723570

[29] Sarris PF, Trantas EA, Baltrus DA, Bull CT, Wechter WP, Yan S, et al Comparative genomics of multiple strains of *Pseudomonas cannabina* pv. *alisalensis*, a potential model pathogen of both monocots and dicots. PLoS ONE. 2013; 8: e59366 10.1371/journal.pone.0059366 23555661 PMC3610874

[30] Polz MF, Alm EJ, Hanage WP. Horizontal gene transfer and the evolution of bacterial and archeal population structure. Trends Genet. 2013; 29: 170–175. 10.1016/j.tig.2012.12.006 23332119 PMC3760709

[31] O'Brien H, Thakur S, Gong Y, Fung P, Zhang J, Yuan L., et al Extensive remodelling of the *Pseudomonas syringae* pv. *avellanae* type III secretome associated with two independent host shifts onto hazelnut. BMC Microbiol. 2012; 12: 141 10.1186/1471-2180-12-141 22800299 PMC3411506

[32] Guttman DS, Mc Hardy AC, Schulze-Lefert P. Microbial genome-enabled insights into plant-microorganism interactions. Nat Rev Genet. 2014; 15: 797–813. 10.1038/nrg3748 25266034

[33] Delcher AL, Bratke KA, Powers EC, Salzberg SL. Identifying bacterial genes and endosymbiont DNA with Glimmer. Bioinformatics. 2007; 23: 673–679. 17237039 10.1093/bioinformatics/btm009 PMC2387122

[34] Altschul SF, Madden TL, Schäffer AA, Zhang Z, Miller W, Lipman DJ. Gapped BLAST and PSI-BLAST: a new generation of protein database search programs. Nucleic Acids Res. 1997; 25: 3389–3402. 9254694 10.1093/nar/25.17.3389 PMC146917

[35] Delcher AL, Phillippy A, Carlton J, Salzberg SL. Fast algorithms for large-scale genome alignment and comparison. Nucleic Acids Res. 2002; 30: 2478–2483. 12034836 10.1093/nar/30.11.2478 PMC117189

[36] Richter M, Rosselló-Móra R. Shifting the genomic gold standard for the prokaryotic species definition. Proc Natl Acad Sci U.S.A. 2009; 106: 19126–19131. 10.1073/pnas.0906412106 19855009 PMC2776425

[37] Guindon S, Gascuel O. A simple, fast and accurate algorithm to estimate large phylogenies by maximum likelihood. Syst Biol. 2003; 52: 696–704. 14530136 10.1080/10635150390235520

[38] Paradis E, Claude J, Strimmer K. APE: analyses of phylogenetics and evolution in R language. Bioinformatics. 2004; 20: 289–290. 14734327 10.1093/bioinformatics/btg412

[39] Huson DH, Bryant D. Application of phylogenetic networks in evolutionary studies. Mol Biol Evol. 2006; 23: 254–267. 16221896 10.1093/molbev/msj030

[40] Wall DP, De Luca T. Ortholog detection using the reciprocal smallest distance algorithm. In: Bergman NH (editor). Methods in Molecular Biology, vol. 396: Comparative genomics, vol. 2. Humana Press Inc., Totowa, U.S.A. 2003 pp. 95–110. 10.1007/978-1-59745-515-2_7 18025688

[41] Poptsova MS, Gogarten JP. BranchClust: a phylogenetic algorithm for selecting gene families. BMC Bioinformatics. 2007; 8: 120 17425803 10.1186/1471-2105-8-120 PMC1853112

[42] Kosakovsky Pond SL, Posada D, Gravenor MB, Woelk CH, Frost SDW. GARD: a genetic algorithm for recombination detection. Bioinformatics. 2006; 22: 3096–3098. 17110367 10.1093/bioinformatics/btl474

[43] Didelot X, Falush D. Inference of bacterial microevolution using mulitlocus sequence data. Genetics. 2007; 175: 1251–1266. 17151252 10.1534/genetics.106.063305 PMC1840087

[44] Rozas J, Sánchez-Del Barrio JC, Messeguer X, Rozas R. DnaSP, DNA polymorphism analyses by the coalescent and other methods. Bioinformatics. 2003; 19: 2496–2497. 14668244 10.1093/bioinformatics/btg359

[45] Tibayrenc M, Ayala FJ. Reproductive clonality of pathogens: a perspective on pathogenic viruses, bacteria, fungi and parasitic protozoa. Proc Natl Acad Sci U.S.A. 2012; 109: E3305–E3313. 10.1073/pnas.1212452109 22949662 PMC3511763

[46] Drummond AJ, Suchard MA, Xie D, Rambaut A. Bayesian phylogenetics with BEAUti and BEAST 1.7. Mol Biol Evol. 2012; 29: 1969–1973. 10.1093/molbev/mss075 22367748 PMC3408070

[47] Ochman H, Wilson AC. Evolution in bacteria: evidence for a universal substitution rate in cellular genomes. J Mol Evol. 1987; 26: 74–86. 3125340 10.1007/BF02111283

[48] Nübel U, Dordel J, Kurt K, Strommenger B, Westh H, Shukla SK, et al (2010) A timescale for evolution, population expansion, and spatial spread of an emerging clone of methicillin-resistant *Staphylococus aureus* . PLoS Pathog. 2010; 8: e1000855. 10.1371/journal.ppat.1000855 PMC2851736 20386717

[49] Ou H-Y, He X, Harrison EM, Kulasekara BR, Thani AB, Kadioglu A, et al Mobilome FINDER: web-based tools for *in silico* and experimental discovery of bacterial genomic islands. Nucleic Acids Res. 2007; 35: Web Server issue, W97–W104. 17537813 10.1093/nar/gkm380 PMC1933208

[50] Overbeek R, Olson R, Pusch GD, Olsen GJ, Davis JJ, Disz T, et al The SEED and the rapid annotation of microbial genomes using subsystems technology (RAST). Nucleic Acids Res. 2014; 42 (database issue): D206–D214. 10.1093/nar/gkt1226 24293654 PMC3965101

[51] Grissa I, Vergnaud G, Pourcel V. CRISPRFinder: a web tool to identify clustered regularly interspaced short palindromic repeats. Nucleic Acids Res. 2007; 35 (Web Server issue): W52–W57. 17537822 10.1093/nar/gkm360 PMC1933234

[52] Nowell RW, Green S, Laue BE, Sharp PM. The extent of genome flux and its role in the differentiation of bacterial lineages. Genome Biol Evol. 2014; 6: 1514–1429. 10.1093/gbe/evu123 24923323 PMC4079204

[53] Forest F, Savolainen V, Chase WV, Lupia R, Bruneau A, Crane PR. Teasing apart molecular versus fossil-based error estimates when dating phylogenetic trees: a case study in the birch family (Betulaceae). Syst Bot. 2005; 30: 118–133.

[54] Jackson RW, Vinatzer B, Arnold DL, Dorus S, Murillo J. The influence of the accessory genome on bacterial pathogen evolution. Mobile Genetic Elements. 2011; 1: 1–11. 22016845 10.4161/mge.1.1.16432 PMC3190274

[55] Alfano JR, Charkowski AO, Deng W-L, Badel JL, Petnicki-Ocwieja T, Van Dijk K, et al The *Pseudomonas syringae* Hrp pathogenicity island has a tripartite mosaic structure composed of a cluster of type III secretion genes bounded by exchangeable effector and conserved effector loci that contribute to parasitic fitness and pathogenicity in plants. Proc Natl Acad Sci U.S.A. 2000; 97: 4856–4861. 10781092 10.1073/pnas.97.9.4856 PMC18322

[56] Sohn KH, Jones JDG, Studholme DJ. Draft genome sequence of *Pseudomonas syringae* pathovar syringae strain FF5, causal agent of stem pit dieback on ornamental pear. J Bacteriol. 2012; 194: 3733–3734. 10.1128/JB.00567-12 22740663 PMC3393499

[57] Arrebola E, Cazorla FM, Codina JC, Gutiérrez-Barranquero JA, Pérez-García A, De Vicente A. Contribution of mangotoxin to the virulence and epiphytic fitness of *Pseudomonas syringae* pv. *syringae* . Int Microbiol. 2009; 12: 87–95. 19784928

[58] Carrión VJ, Gutiérrez-Barranquero JA, Arrebola E, Bardaji L, Codina JC, De Vicente A, et al The mangotoxin biosynthetic operon (mbo) is specifically distributed within *Pseudomonas syringae* genomospecies 1 and was acquired only once during evolution. Appl Environ Microbiol. 2013; 79: 756–767. 10.1128/AEM.03007-12 23144138 PMC3568555

[59] Carrión VJ, Arrebola E, Cazorla FM, Murillo J, de Vicente A. The *mbo* operon is specific and essential for biosynthesis of mangotoxin in *Pseudomonas syringae* . PLoS ONE. 2012; 7: e36709 10.1371/journal.pone.0036709 22615797 PMC3355146

[60] Carrión VJ, Van der Voort M, Arrebola E, Gutiérrez-Barranquero JA, De Vicente A, Raaijmakers JM et al Mangotoxin production of *Pseudomonas syringae* pv. syringae is regulated by MgoA. BMC Microbiol. 2014; 14: 46 10.1186/1471-2180-14-46 24555804 PMC3945005

[61] Ortega MA, Hao Y, Zhang Q, Walker MC, Van der Donk VA, Nair SK. Structure and mechanism of the tRNA-dependent lantibiotic dehydratase NysB. Nature. 2015; 517: 509–512. 10.1038/nature13888 25363770 PMC4430201

[62] Sahl HG, Bierbaum G. Lantibiotics: biosynthesis and biological activities of uniquely modified peptides from gram-positive bacteria. Annu Rev Microbiol. 1998; 52: 41–79. 9891793 10.1146/annurev.micro.52.1.41

[63] Cotter PD, Ross RP, Hill C. Bacteriocins: a viable alternative to antibiotics? Nat Rev Microbiol. 2013; 11: 95–105. 10.1038/nrmicro2937 23268227

[64] Sano Y, Kageyama M. A novel transposon-like structure carries the genes for pyocin AP41, a *Pseudomonas aeruginosa* bacteriocin with a DNase domain homology to E2 group colicins. Mol Gen Genet. 1993; 237: 161–170. 8384291 10.1007/BF00282797

[65] Buell CR, Joardar V, Lindeberg M, Selengut J, Paulsen IT, Gwinn ML, et al The complete genome sequence of the *Arabidopsis* and tomato pathogen *Pseudomonas syringae* pv. *tomato* DC3000. Proc Natl Acad Sci U.S.A. 2003; 100: 10181–10186. 12928499 10.1073/pnas.1731982100 PMC193536

[66] Joardar V, Lindeberg M, Jackson RW, Selengut J, Dodson R, Brinkac LM, et al Whole-genome sequence analysis of *Pseudomonas syringae* pv. phaseolicola 1448A reveals divergence among pathovars in genes involved in virulence and transposition. J Bacteriol. 2005; 187: 6488–6498. 16159782 10.1128/JB.187.18.6488-6498.2005 PMC1236638

[67] Bender CL, Cooksey DA. Indigenous plasmids in *Pseudomonas syringae* pv. tomato: conjugative transfer and role in copper-resistance. J Bacteriol. 1986; 165, 534–541. 3003029 10.1128/jb.165.2.534-541.1986 PMC214452

[68] Bender CL, Cooksey DA. Molecular cloning of copper resistance genes from *Pseudomonas syringae* pv. tomato. J Bacteriol. 1987; 169: 470–474. 3027030 10.1128/jb.169.2.470-474.1987 PMC211800

[69] Nakajima M, Goto M, Hibi T. Similarity between copper resistance genes from *Pseudomonas syringae* pv. *actinidiae* and *P*. *syringae* pv. *tomato* . J Gen Plant Pathol. 2002; 68: 68–74.

[70] Cooksey AD, Azad HR, Cha JS, Lim CK. Copper resistance gene homologs in pathogenic and saprophytic bacterial species from tomato. Appl Environ Microbiol. 1990; 56: 431–435. 16348118 10.1128/aem.56.2.431-435.1990 PMC183357

[71] Bultreys A, Gheysen I, De Hoffman E. Yersiniabactin production by *Pseudomonas syringae* and *Escherichia coli*, and description of a second yersiniabactin locus evolutionary group. Appl Environ Microbiol. 2006; 72: 3814–3825. 16751485 10.1128/AEM.00119-06 PMC1489633

[72] Chaturvedi KS, Hung CS, Crowley JR, Stapleton AE, Henederson JP The siderophore yersiniabactin binds copper to protect pathogens during infection. Nat Chem Biol. 2012; 8: 731–736. 10.1038/nchembio.1020 22772152 PMC3600419

[73] Bauer DW, Collmer A. Molecular cloning, characterization, and mutagenesis of a *pel* gene from *Pseudomonas syringae* pv. *lachyrmans* encoding a member of the *Erwinia chrysanthemi* pelADE family of pectate lyases. Mol Plant Microbe Interact. 1997; 10: 369–379. 9100381 10.1094/MPMI.1997.10.3.369

[74] Liao CH, Fett W, Tzean SS, Hoffman G. Detection and sequence analysis of an altered pectate lyase gene in *Pseudomonas syringae* pv. *glycinea* and related bacteria. Can J Microbiol. 2006; 52: 1051–1059. 17215896 10.1139/w06-063

[75] Khandekar S, Srivastava A, Pletzer D, Stahl A, Ullrich MS. The conserved upstream region *of lscB/C* determines expression of different levansucrase genes in plant pathogen *Pseudomonas syringae* . BMC Microbiol. 2014; 14: 79 10.1186/1471-2180-14-79 24670199 PMC3973379

[76] Denny T. Involvement of bacterial polysaccharides in plant pathogenesis. Annu Rev Phytopathol. 1995; 33: 173–197. 18999958 10.1146/annurev.py.33.090195.001133

[77] Galeva A, Moroz N, Yoon Y-H, Hughes KT, Samatey FA, Kostyukova AS. Bacterial flagellin-specific chaperone FliS interacts with anti-sigma factor FlgM. J Bacteriol. 2014; 196: 1215–1221. 10.1128/JB.01278-13 24415724 PMC3957722

[78] Newman M-A, Sundelin T, Nielsen JT, Erbs G. MAMP (microbe-associated molecular pattern) triggered immunity in plants. Front Plant Sci. 2013; 4: 139 10.3389/fpls.2013.00139 23720666 PMC3655273

[79] Péchy-Tarr M, Bruck DJ, Maurhofer M, Fischer E, Vogne C, Henkels MD, et al Molecular analysis of a novel gene cluster encoding an insect toxin in plant-associated strains of *Pseudomonas fluorescens* . Environ Microbiol. 2008; 10: 2368–2386. 10.1111/j.1462-2920.2008.01662.x 18484997

[80] Shrivastava S, Mande SS. Identification and functional characterization of gene components of type VI secretion system in bacterial genomes. PLoS ONE 2008; 3: e2955 10.1371/journal.pone.0002955 18698408 PMC2492809

[81] Mattick JS. Type IV pili and twitching motility. Annu Rev Microbiol. 2002; 56: 289–314. 12142488 10.1146/annurev.micro.56.012302.160938

[82] Jin F, Conrad JC, Gibiansky ML, Wong GCL. Bacteria use type-IV pili to slingshot on surfaces. Proc Natl Acad Sci U.S.A. 2011; 108: 12617–12622. 10.1073/pnas.1105073108 21768344 PMC3150923

[83] Hacker J, Carniel E. Ecological fitness, genomic islands and bacterial pathogenicity. A Darwinian view of the evolution of microbes. EMBO Rep. 2001; 2: 376–381. 11375927 10.1093/embo-reports/kve097 PMC1083891

[84] Yeaman S. Genomic rearrangements and the evolution of clusters of locally adaptive loci. Proc Natl Acad Sci U.S.A. 2013; 110: E1743–E1751. 10.1073/pnas.1219381110 23610436 PMC3651494

[85] Freeling M, Lyons E, Pedersen B, Alam M, Ming R, Lisch D. Many or most genes in *Arabidopsis* transposed after the origin of the order Brassicales. Genome Res. 2008; 18: 1924–1937. 10.1101/gr.081026.108 18836034 PMC2593585

