# Supplementary figures and images for: Comparative Genomic Analyses of Multiple Pseudomonas Strains Infecting Corylus avellana Trees Reveal the Occurrence of Two Genetic Clusters with Both Common and Distinctive Virulence and Fitness Traits
Source: PLoS One. 2015 Jul 6;10(7):e0131112. doi: 10.1371/journal.pone.0131112 (PMC4492584; doi:10.1371/journal.pone.0131112)

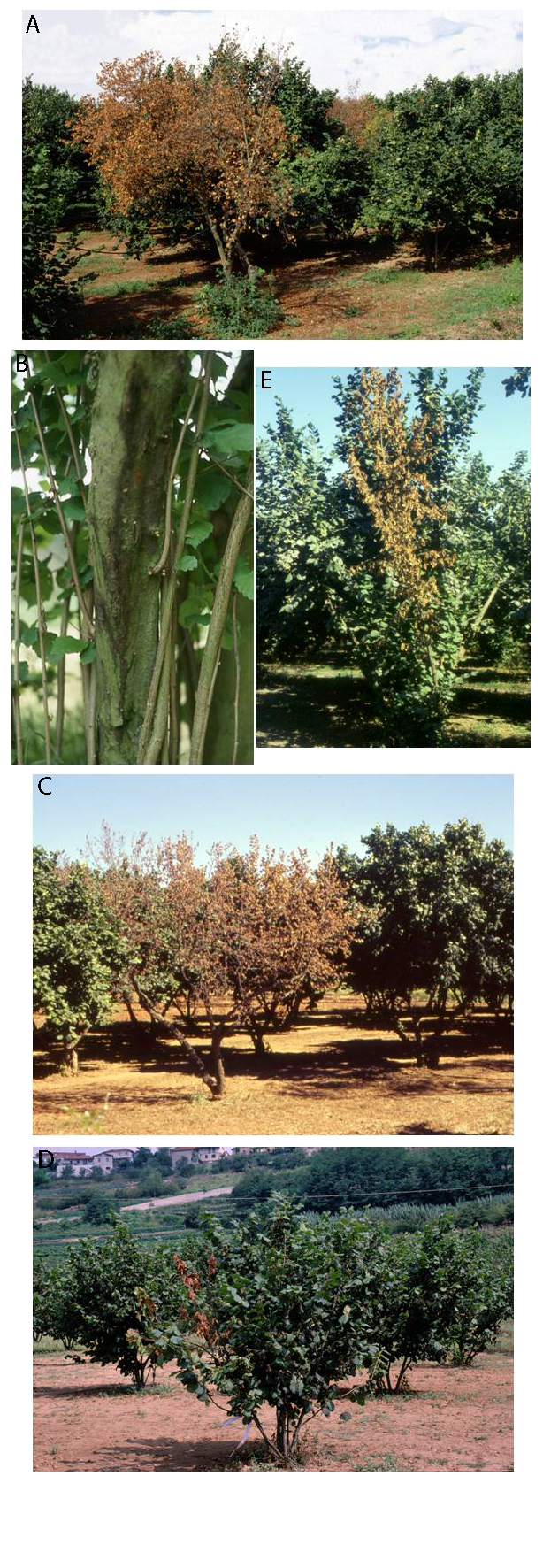

Supplement: S1 Fig — A) tree death caused by P. avellanae; B) longitudinal canker on trunk incited P. avellanae; C) tree death caused by P. syringae pv. avellanae; D) twig wilting caused by P. s. pv. coryli; E) branch die-back incited by P. s. pv. syringae. (TIF) [file pone.0131112.s001.tif]

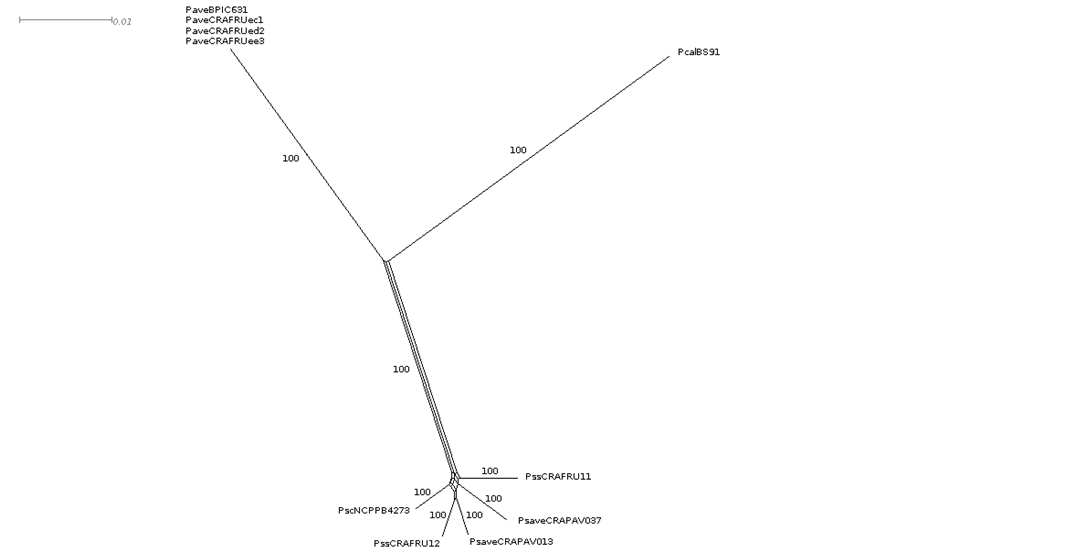

Supplement: S2 Fig — Tree based on split analysis of gapA, gltA, gyrB and rpoD, for a total of 6.812 nucleotides of Pseudomonas avellanae and P. syringae pathovars avellanae, coryli and syringae infecting Corylus avellana trees. Bootstrap values are shown at the main nodes. The scale bar indicates the number of substitutions per nucleotide position. Strain legend is shown in Table 1. P. cannabina pv. alisalensis Pcal BS91 was included in the analysis as outgroup. (TIF) [file pone.0131112.s002.tif]

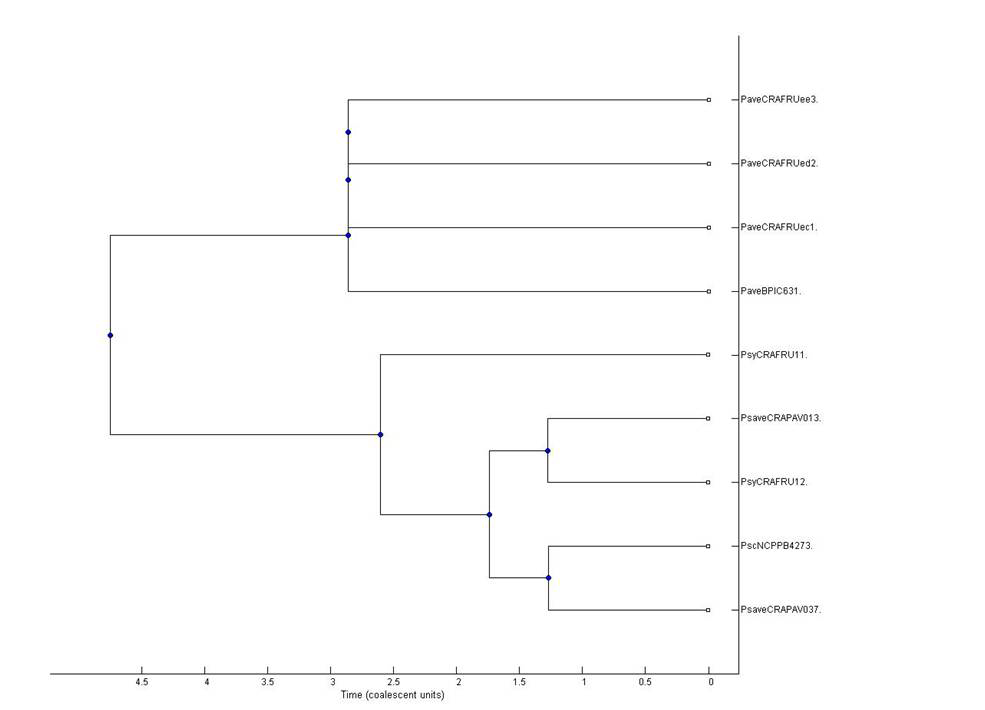

Supplement: S3 Fig — The tree is based on the partial sequence of four housekeeping genes (i.e., gapA, gltA, gyrB, rpoD). Strain legend is shown in Table 1. (TIF) [file pone.0131112.s003.tif]

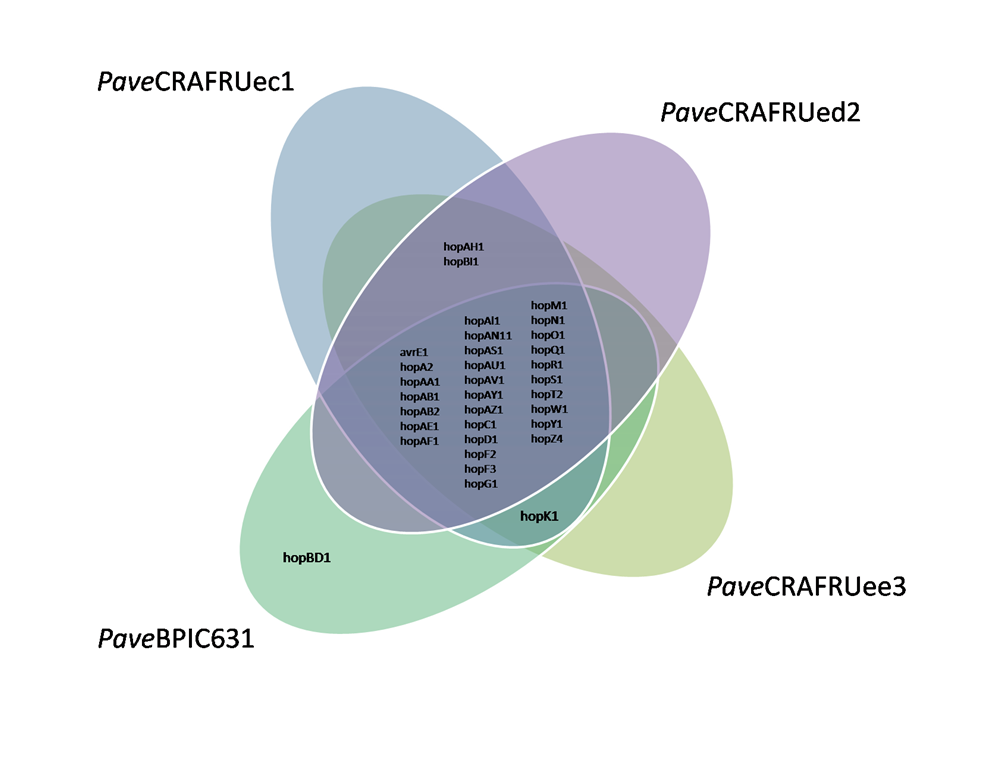

Supplement: S4 Fig — Strain legend is shown in Table 1. (TIF) [file pone.0131112.s004.tif]

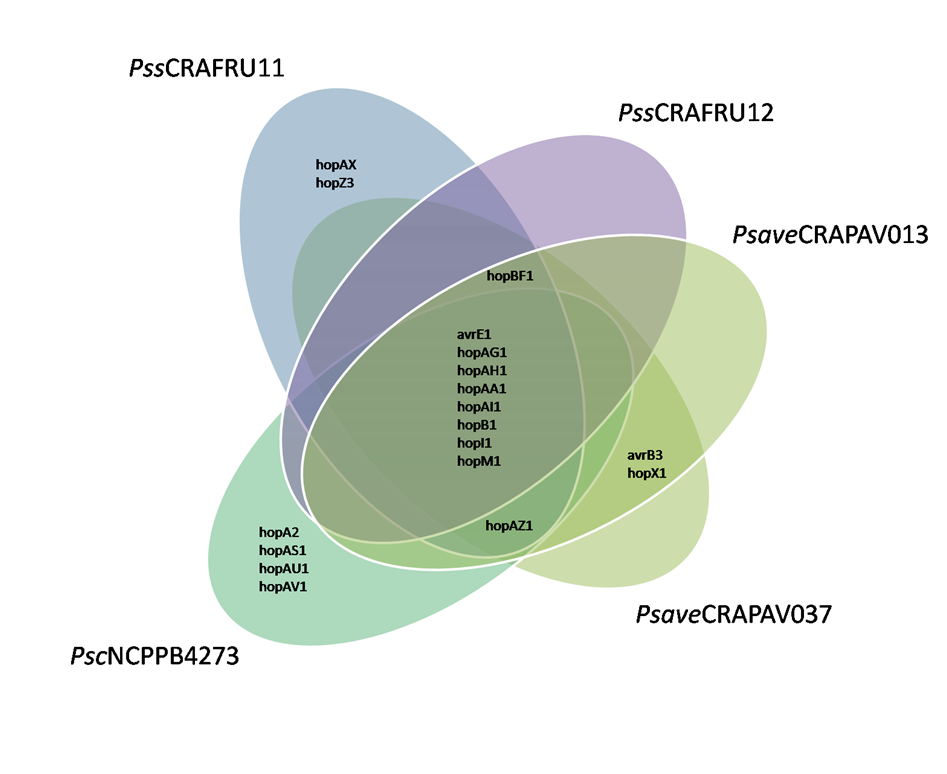

Supplement: S5 Fig — Strain legend is shown in Table 1. (TIF) [file pone.0131112.s005.tif]

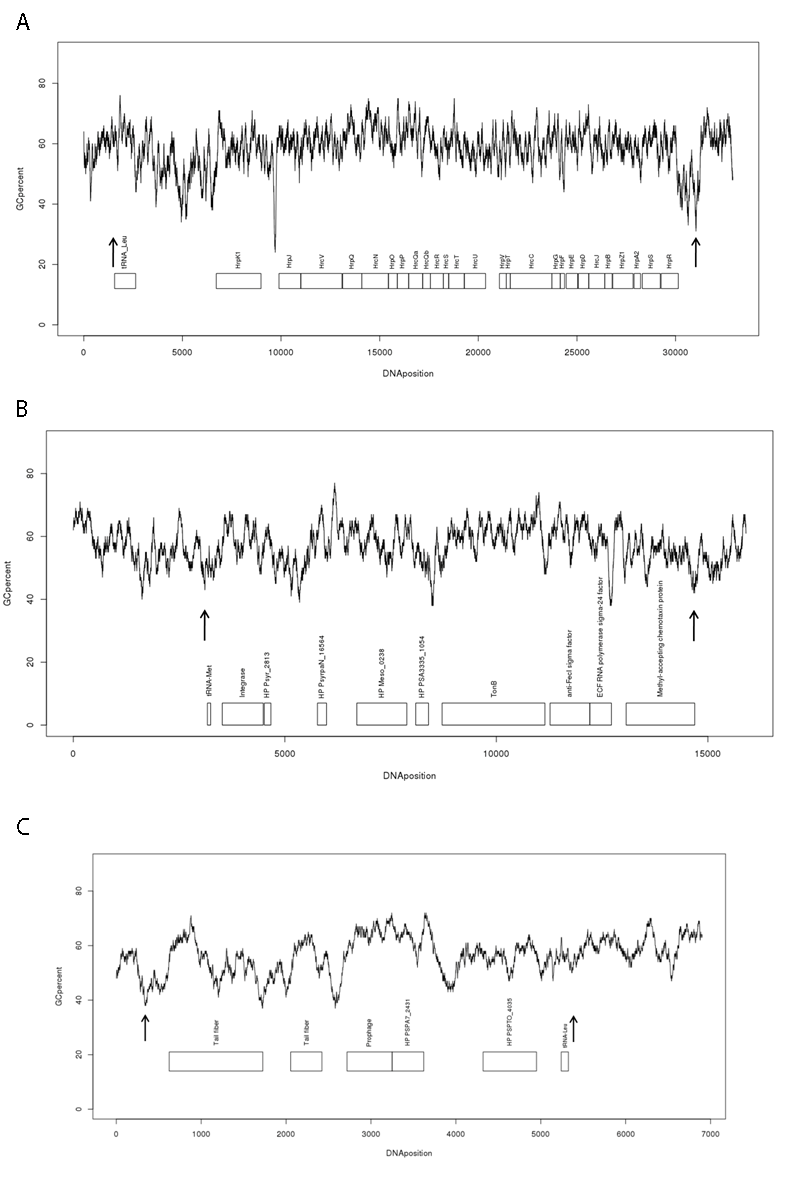

Supplement: S7 Fig — It was obtained using R software. Arrows indicate the beginning and the end of the island. Putative hortolog genes are indicated. A) Pcor GI 1, identified in all pseudomonad strains infecting C. avellana; B) and C) Pave GI2 and Pave GI3, respectively, identified solely in the Pseudomonas avellanae strains. (TIF) [file pone.0131112.s007.tif]

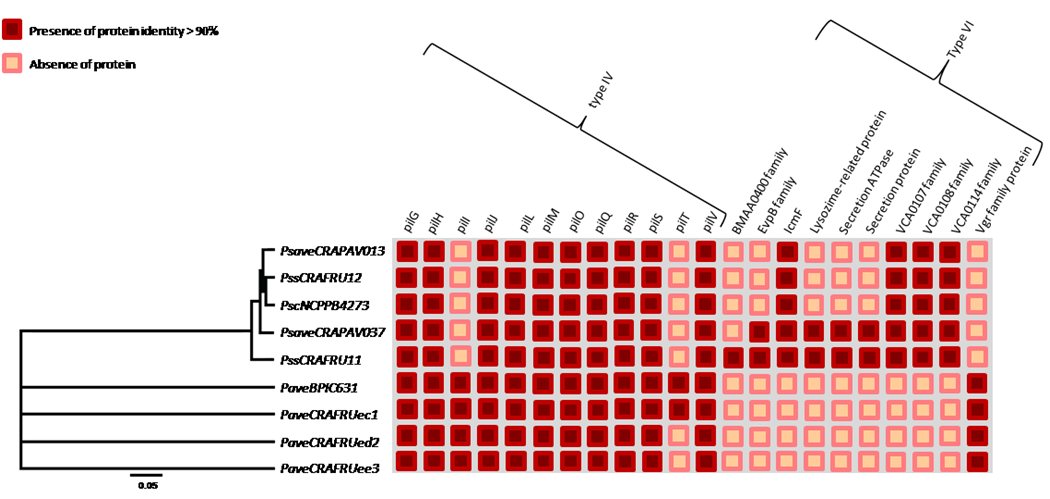

Supplement: S8 Fig — The indication concerns the presence of type IV and type VI secretion systems homologues. Strain legend is shown in Table 1. (TIF) [file pone.0131112.s008.tif]
