# Supplementary material for: Comparative Genomic Analyses of Multiple Pseudomonas Strains Infecting Corylus avellana Trees Reveal the Occurrence of Two Genetic Clusters with Both Common and Distinctive Virulence and Fitness Traits
Source: PLoS One. 2015 Jul 6;10(7):e0131112. doi: 10.1371/journal.pone.0131112 (PMC4492584; doi:10.1371/journal.pone.0131112)
Supplement: S1 Table — (DOC) [file pone.0131112.s009.doc]

Table S1.

Twig dieback Trunk canker Tree death

*Pseudomonas avellanae* +++ +++ +++

*P. syringae* pv. *avellanae* +++ ++ ++

*P. syringae* pv. *coryli*  ++ + -

*P. syringae* pv. *syringae* + + -

Table S2.

| Gene number | Gene name |
| --- | --- |
| 1 | *hrcS* |
| 2 | *hrpS* |
| 3 | *hrcR* |
| 4 | *hrcT* |
| 5 | *hrcQb* |
| 6 | *hrcU* |
| 7 | *hrcQa* |
| 8 | *hrpP* |
| 9 | *hrpV* |
| 10 | *hrpO* |
| 11 | *hrpT* |
| 12 | *hrcN* |
| 13 | *hrcC* |
| 14 | *hrcQ* |
| 15 | *hrpG* |
| 16 | *hrpF* |
| 17 | *hrpE* |
| 18 | *hrcV* |
| 19 | *hrpD* |
| 20 | *hrcJ* |
| 21 | *hrpB* |
| 22 | *hrpJ* |
| 23 | *hrpZ1* |
| 24 | *hrpR* |
| 25 | *hrpA2* |
